# Supplementary material for: Marine spatial planning makes room for offshore aquaculture in crowded coastal waters
Source: Nat Commun. 2018 Mar 5;9:945. doi: 10.1038/s41467-018-03249-1 (PMC5838171; doi:10.1038/s41467-018-03249-1)
Supplement: Supplementary file 1 — Supplementary Information [file 41467_2018_3249_MOESM1_ESM.pdf]

Supplementary Notes

An overview of the key steps for implementing the full MSP analytical model, including the significance of each step and a summary of how each step was implemented within this case study analysis, is found in Supplementary Table 1. In the following supplementary notes, we provide additional details about the component models used in our case study analysis.

Supplementary Note 1.

Ocean Circulation Model: A three-dimensional ocean circulation model (OCM) contributed to multiple parts of this study. The OCM is a high-resolution Regional Ocean Modeling System (ROMS) applied to the SCB region[1,2]. Implemented by Dong and McWilliams[2], the OCM is driven by realistic boundary conditions extracted from a nested ROMS solution for the U.S. West Coast with high-resolution air-sea forcing. Detailed information on the lateral and surface boundary conditions and model validation can be found in Dong and McWilliams[2] and Dong et al.[3]. The OCM has a 1-km horizontal grid and 40 vertical levels and covers the same area as the study domain. Results from the OCM consist of three-dimensional flow fields and temperature and two-dimensional mixed layer depth, which contributed to the aquaculture models, the larval dispersal component of the halibut fishery model, and the disease model. For the halibut model (Supplementary Note 9) and the disease risk model (Supplementary Note 13), the OCM was run from 1996-2002. For the aquaculture models, the OCM was run using environmental data from 2000 to 2001. This time period was chosen because it was a neutral-condition El Niño Southern Oscillation (ENSO) period[4], and thus represents “average” oceanographic conditions.

Supplementary Note 2.

Introduction to Aquaculture Production and Cost Models: To estimate the value of the three types of aquaculture farms (mussel, finfish, and kelp) we developed separate spatially-explicit bioeconomic models for each, then evaluated the models for sites that met the fixed constraints to aquaculture development. Each aquaculture model contains a production and a cost model. Production models estimate annual yield of a given aquaculture type (given a specified farm design for each type of aquaculture, described in detail below) within a site based on environmental conditions in that location (e.g. water temperature, currents, productivity, etc.). We then multiply that yield by a market price to determine annual revenue. Cost models incorporate effects of environmental conditions (e.g., wave height, depth) and geographic location (e.g., distance from port, depth) on operational and maintenance costs of the farm. Farm designs and cost estimates were based on aquaculture development plans from industry, which we then scaled to a 1-km^2 farm size. When necessary to respect confidentiality, we only report aggregated cost data. Additionally, much of the economic information provided by industry was preliminary, outdated and/or highly aggregated. Therefore, while the exact cost numbers are uncertain, they are internally consistent within an aquaculture type and thus are appropriate for evaluating spatial variation in aquaculture value. Given that offshore aquaculture is a relatively new industry (particularly for finfish), we expect significant technological improvements and knowledge acquisition over the coming years that are likely to change the productive output and operational costs of aquaculture. As for many new industries, we expect that in most cases these developments will make the industry more productive and more cost-effective, increasing the number of sites that would be profitable and providing a larger number of options for developing aquaculture while minimizing impacts and tradeoffs. Our models can be adjusted for these new

scenarios in the future, and because of the rapidly evolving nature of the industry, we focus on spatial variation in productivity and profitability rather than absolute numbers.

For all farms, we assumed that costs (e.g., of fuel, labor) would not change over the 10-year evaluation period and that the farm equipment has a lifespan of at least 10 years. Since there is currently no cost for leases in federal waters, we excluded any lease cost in our models. There are costs associated with a lease in state waters[5], but we treated all sites equally in this respect so as to not bias development in favor of federal water sites. Deeper sites may have a larger bottom footprint due to the anchoring system design and therefore may be more difficult and costly to permit – issues that we do not explicitly address in our model. Some costs, especially administrative and maintenance costs, could decrease if the same company had farms in multiple sites, but we assumed that each farm is a separate, individually functioning entity and did not consider economies of scale.

For each aquaculture type, sites with negative value (NPV and annuity) were assumed to be undevelopable. These economic constraints, in addition to the logistical and regulatory spatial constraints (see Methods in main paper), restricted aquaculture development to 1,061 sites (Supplementary Fig. 1; 1,011 for mussel, 392 for finfish, and 325 for kelp). All calculations, unless otherwise indicated, were conducted using Microsoft Excel or MATLAB (MATLAB and Statistics Toolbox Release 2013a, The MathWorks, Inc., Natick, Massachusetts, United States).

Supplementary Note 3.

Mussel Aquaculture Production Model: The layout of modeled mussel farms was developed to reflect a feasible farm design for a 1-km^2 area based on industry practice within the Southern California region (Pers. Comm., B. Friedman, Santa Barbara Mariculture; Pers. Comm. P. Cruver, Catalina Sea Ranch). Specifically, each farm contains 100 longlines, each 210 m long and spaced 30 m apart. Each longline has 3,962 meters of fuzzy rope to which individual juvenile mussels are attached for grow-out; 328 individual mussels are seeded per meter of fuzzy rope. Thus, each farm consists of ~130,000,000 individual mussels. Growth of individuals in the production model continues until the summed weight of all individuals reaches 2,948,350 kg (an average of 0.023 kg per whole mussel), at which point a harvesting event occurs. Harvesting was assumed to be continuous throughout the year, meaning that lines are re-stocked immediately following harvest. We assumed farms can operate at full capacity every year over the 10-year evaluation period. Average annual yield for a farm was multiplied by an assumed wholesale price of farmed mussels of $3.30/kg to estimate the farm's annual revenue (Supplementary Fig. 2a). The wholesale price was approximated based on informal discussions with industry representatives, and is consistent with the ex-vessel value of *Mytlius edulis* landings in 2016 of $3.71/kg based on National Marine Fisheries Service commercial landings statistics (https://www.st.nmfs.noaa.gov/commercial-fisheries/commercial-landings/annual-landings/index).

We used a dynamic energy budget model (DEB) to predict mussel growth[6]. DEB models have been used in ecological literature to describe energy fluxes in individuals, and have previously been applied to the growth of species under aquaculture conditions[7-9]. We parameterized the model based on the Mediterranean mussel (*Mytilus galloprovincialis*), since this is the only commercially grown mussel in southern California and is the most likely target species for future shellfish aquaculture development in the region[10]. The DEB model describes the rates at which an individual mussel consumes food and uses energy for growth, somatic maintenance,

reproduction, and development. We assumed no natural re-seeding of the lines from mussel reproduction; spat procurement was included in the farm cost model. The rate of food consumption is primarily dependent on the size of the individual, food availability, and energy requirements at a given time. Growth parameters for the species followed Montalto et al.[11] and Kooijman et al.[12]. Food availability was determined using MODIS and SeaWiFS satellite derived spatial data to estimate the particulate organic carbon (POC) concentration in each developable site[13-15]. The OCM (Supplementary Note 1) also provided model inputs for each developable site including monthly average surface temperature, mixed layer depth, and current magnitude in the mixed layer, over years 2000 and 2001 neutral-condition El Niño Southern Oscillation years; [4]. See Supplementary Data 2 for a full list of model parameters.

The DEB model developed by Muller and Nisbet[6] was extrapolated to estimate the potential yield of an entire mussel farm. The DEB model estimates individual mussel growth as a function of food-carbon availability (for our purposes POC, measured in mgC). To apply this model to all mussels on a farm, we first modeled the dynamics of food availability within the entire farm array using a simple box-model approach. Using this method, the dynamics of food availability (and consequently mussel growth) are characterized by the input supply of POC into the volume occupied by the farm (the 'box') from the surrounding ocean, the consumption of POC by mussels within the farm, and the flux of POC out of the farm into the surrounding ocean. Next we made the following assumptions: 1) all mussels experience the same food availability regardless of position in the farm array, 2) POC concentration is uniform in the mixed layer, and 3) flux of water is constant throughout the volume occupied by the farm. Under these assumptions, the individual DEB model of mussel growth was applied to each mussel in the fixed-design farm array described above. The box-model of POC dynamics within the mussel farm is described as follows.

Under assumption (2) above, the rate of POC supply (in mgC s^{-1}), F_{in}, is calculated as the surface concentration of POC (in mgC m^{-3}), $X_{c(0)}$, multiplied by the flux of water (m^3 s^{-1}) entering the farm, r,

$$F_{in} = X_{c(0)} r \,, \qquad \text{(Sup Equ. 1)}$$

Where r is calculated as current speed, V (measured in m s^{-1}), multiplied by the cross-sectional area of the farm (measured in m^2), F_{area},

$$r = V F_{area} \,, \qquad \text{(Sup Equ. 2)}$$

And the cross-sectional area of the farm is computed as the width, W, of the site in which the farm is located (1,000m for 1km^2 sites) multiplied by the mixed layer, mld.

$$F_{area} = W mld \,, \qquad \text{(Sup Equ. 3)}$$

Under assumption (3) above, F_{in} describes the rate of POC supply within each 'F_{area} x 1m' volume of the farm (in units of mgC). Given that the length of each site is 1000 m, the total POC input supply rate (in mgC s^{-1}) into the volume occupied by the entire farm array is 1000 x F_{in}.

The population of mussels within a given farm then consume available POC at a rate determined by,

$$C = nJ_x \,, \qquad \text{(Sup Equ. 4)}$$

where n is the number of mussels within a farm, and J_x is the scaled individual rate of food consumption, in mgC s^{-1} (parameter values and references are listed in Supplementary Data 2). Unconsumed POC then flows out of the farm at a rate, X_c, determined by mass balance at time (t). Therefore, under assumption (1), and using Sup Equ. 1-3, the dynamics of food availability over time (t) within the farm are governed by Sup Equ. 5, which states that the rate of change in total POC is the rate of supply minus the rate of consumption and the rate of outflow.

$$\frac{\partial POC}{\partial t} = 1000 X_{c(0)} r(t) - nJ_x - 1000 X_{c(t)} r(t) \,, \qquad \text{(Sup Equ. 5)}$$

Given this transition equation and the requisite input data (surface POC concentration, mixed layer depth, current speed), as well as mixed layer temperature (which is used in the DEB model), the time path of POC completely determines individual mussel growth, which (under all the previously stated assumptions) also completely determines farm-level yield and revenues.

Supplementary Note 4.

Mussel Aquaculture Cost Model: We developed a mussel cost model based on various industry projections for each 1-km^2 farm site that included both starting costs (including construction and equipment costs for the farm and hatchery) and annual operating costs (Pers. Comm., B. Friedman, Santa Barbara Mariculture; Pers. Comm. P. Cruver, Catalina Sea Ranch). Starting costs were assumed to be constant across locations and only incurred during the first year of production. Annual operating costs included two categories: fixed operating costs that are not sensitive to location, including vessel maintenance, vessel docking, and monitoring costs, and variable costs that vary in relation to farm location (described below). Fixed and variable operating costs then were combined into a single annual operating cost for each site and were combined with the starting costs in year one to estimate total average annual cost of the farm (Supplementary Fig. 3a). Annual revenue and costs were then used to calculate NPV and equivalent annuities (Eq. 1-2).

Variable (i.e. location-specific) operating costs, including fuel, labor, operations and maintenance costs, were estimated as follows. Fuel used for transport to the farm site was adjusted to account for distance from port. Locations of major fishing ports (San Diego, Mission Bay, Oceanside, Dana Point, Newport Beach, Long Beach, Redondo Beach, Marina del Rey, Port Hueneme/Channel Islands Harbor, Ventura, Santa Barbara, and Avalon on Catalina Island)[16] were digitized using Google Earth and their point locations imported into ArcGIS 10.2 (Supplementary Data 1). Distance to port was calculated for each site in the planning grid using the ArcGIS 10.2 Cost Distance tool, which calculates the distance to the nearest source (port) for each site in the raster, based on the least-accumulative cost over a cost surface (in this case, over-water sites are equally weighted and over-land sites are excluded so that travel is required to go around islands and headlands). We assumed two identical farm boats making trips to each farm: one going 5 days a week and one 3 days a week, resulting in 416 round trips to the farm site each year. Annual fuel costs (AFC) were thus approximated as:

$$AFC = \frac{TD_{port}F_eP_f}{s}, \qquad \text{(Sup Equ. 6)}$$

where T is the number of trips, D_{port} is the distance from port to the farm, s is the average boat speed, F_e is the fuel efficiency of the boat, and P_f is fuel price; see Supplementary Data 2 for parameter values.

Labor costs, L_t, were adjusted to account for the extra time it would take for transport to the farm site based on distance from port. We assumed that labor for each farm requires 8 workers to visit and service the farm 5 days a week, totaling 2080 worker days per year, and that each laborer would be paid $11 per hour, including for transport time to get to the farm site. Labor costs thus consist of fixed costs (8 hours per day for 2080 worker days) plus variable costs (transport time for every worker day), with labor costs increasing with distance from port to farm.

Operations and maintenance costs were adjusted to account for the increased costs associated with farming in locations with higher wave energy[17,18]. In order to account for this increased cost, we multiplied on-farm operations and maintenance costs (primarily consisting of labor costs; not including transport, seed, or hatchery costs) by a factor of 1.5 for sites which have a mean significant wave height greater than 1 m. While the exact relationship between waves and costs is not known and likely varies across operations, we based this estimate on the best available information from industry reports[18,19].

Total costs for on-farm operations and maintenance were also increased by 10% for farms located in greater than 50 meters depth. This cost increase accounts for SCUBA diving depth limits, which would likely result in higher costs for servicing to the anchoring systems or benthic monitoring (which is only a small part of total operational expenses) for farms located in deeper waters. Finally, the operational hatchery and seed costs were multiplied by the average number of growing cycles at each site per year (which depended on the productivity of the location). Taking all of these factors into account, the cost of operations amount to $2,123,576 to $3,219,202 per farm site per year, depending on the site.

Supplementary Note 5.

Kelp Aquaculture Production Model: There are no commercial kelp farms or kelp farm proposals in California, and thus our model of kelp aquaculture in Southern California is based on extrapolations from best estimates in the literature and from kelp aquaculture operations elsewhere. Most of our information on practices and design of kelp farms was informed by the kelp aquaculture industry[20] and a report issued by Irish Sea Fisheries Board on development and demonstration of seaweed aquaculture methodologies[21]. We parameterized our model based on the brown algae (kelp) *Saccharina latissima,* also known as *Laminaria saccharina*. Its farming methods are well known[20], and there is a proven high-end market for this product[22].

To model the growth and biomass production of kelp farms we used a dynamic individual growth model of *S. latissima* developed by Broch and Slagstad[23]. Parameters derived from Feldman and McClain[14] and OCM data were used to vary environmental conditions across sites (see Supplementary Data 2 for full parameter list). Additionally, we assumed that all kelp plants

have access to the same amount of nutrients regardless of their placement within the farm, i.e., growth rates are the same for all individuals within a single farm[24].

We used a similar farm design for the kelp as was used in the mussel production model, with some notable exceptions. We assumed that kelp lines could be grown closer together than mussels, as they are generally cultivated closer to the surface, and do not require fuzzy rope for cultivation. As a result, we assumed that each kelp farm would consist of 200 lines, each 210 m long and set 20 m apart.

The overall start date and end date of a growing season was dependent on the seasonal availability of nitrate in each site. The growing season could potentially start as early as 1 October and end as late as 15 April of each simulated year (197 days). We chose to end the growing season mid-April because encrusting by bryozoans later in the season would likely decrease the value of farmed kelp[25]. Depending on the environmental conditions, the kelp may reach a maximum size before the end of the growing season. In this case, each individual kelp was trimmed by 75% and allowed to continue to grow until the end of the harvest season where it was subject to a final harvest. The trimmed plant area was then treated as harvested biomass and calculated for all sites.

There are two main markets for kelp – as a premium food product and as an ingredient in a diversity of products such as animal feeds and fertilizer, and as an emulsifying or stabilizing additive in products such as cosmetics and ice cream; [26,27]. There are also emerging markets such as the potential use of kelp for biofuels and in certain aquaculture feeds[28]. Currently the market for premium food products brings the highest prices[26]. However, it unclear how big this market is and at what level of production this market would become saturated. The type of operation that we modeled will only be profitable if a premium market is available. As a result we used a fixed market price of $3 per kg for all farms to calculate annual revenue (Supplementary Fig. 2b), acknowledging that this price may not be accurate if the premium market becomes saturated.

Supplementary Note 6.

Kelp Aquaculture Cost Model: Because of similarities in construction and servicing of kelp and mussel farms, the kelp cost model (Supplementary Fig. 3b) was based on the mussel cost model, and unless otherwise noted uses the same parameters, structure, and assumptions. For representing operational procedures particular to kelp farming[20-23], we made the following adjustments to the mussel cost model:

1. Starting costs were adjusted in three ways:

   - *Spacing:* Because kelp lines are placed more closely together than the mussel lines, starting costs were adjusted to account for the increased number of lines per farm.
   - *Longline gear and harvesting equipment reduced*: Loops of fuzzy rope are not needed for kelp farming, thus we eliminated the cost of the fuzzy rope from the equipment costs.
   - *Hatchery costs:* We approximated the start-up seed and hatchery costs based on the best available information. The Irish Sea Fisheries Board estimates the cost of setting up a hatchery to be $45,000[21]. This value was converted to US dollars at the rate of 1.43 dollars/Euro, which was the exchange rate in May of 2011 when the report was published. Since their case study farm is only 1/3 the size of the one used in our model, we multiplied this cost by three in order to represent potential farms in SCB in our model.

2. Labor needs for the farm were divided into the amount of labor required for seeding, maintenance, and harvest. The labor required for seeding was the same at all locations, but the amount of labor days needed for harvest depends on the amount of kelp produced (including final harvest and “trimming”). This harvest labor was calculated at the rate of 4 tons per day per person. The additional labor cost for transport was calculated in the same way as the mussel model.

3. Since our kelp model did not require multiple seeding events in a single year, operational seed and hatchery costs do not vary among farms, and thus were included in fixed rather than variable operational costs.

4. We removed the cost of mussel seed from the operating costs (since all kelp propagation is done in the hatchery).

5. We changed the yearly operational costs of the hatchery (included in fixed operating costs) to reflect the different hatchery process required for kelp. Costs were based on the Irish Sea Fisheries report, which estimates hatchery costs to be $130,871 annually[21]. We multiplied this cost by three (since our farm design is producing approximately three times the production of the Irish farm) to estimate the annual cost of a kelp hatchery.

Supplementary Note 7.

Finfish Aquaculture Production Model: To estimate the production of finfish aquaculture in the SCB, we used AquaModel[29-32], an advanced, proprietary, GIS-based modeling proprietary software package with a track record of being used by several domestic and foreign government agencies to estimate site-specific finfish aquaculture production and the associated benthic and water column environmental effects. AquaModel simulates the growth (based on a Von Bertalanffy growth function) and metabolic activity of cultured fish as well as the three-dimensional flow and transformation of nutrients, oxygen, and particulate wastes in adjacent waters and sediments. The fish growth and physiology components of the model consist of a nutrient budget for carbon, oxygen, and nitrogen, based on functions describing metabolism, ingestion, egestion, assimilation, respiration and growth as determined by the size of the fish, water temperature, dissolved oxygen concentration, swimming speed, feed rate and composition[33]. We focused on striped bass, *Morone saxatilis*, as the farmed species for finfish aquaculture because it has been modeled previously in AquaModel and was being considered for offshore aquaculture in southern California at the time of this study[34,35].

In our model, farm design and cost is based on a previous proposal by Hubbs-SeaWorld Research Institute [34] to develop a farm off the coast of San Diego in the SCB, and informed by personal communication with Hubbs-SeaWorld Research Institute aquaculture experts (Pers. Comm., D. Kent, Hubbs-SeaWorld Research Institute). Within a planning site we modeled one farm consisting of two rows of 25 m long by 25 m wide by 14.4 m deep surface cages in two rows of twelve cages each, for a total of 24 cages. The cages were stocked to a density of 0.2 kg fish per m^3 cage volume, with individual juvenile fish weighing 20 g at the time of stocking. Fish are then grown in the cages for 18 months (‘grow-out period’). Environmental inputs into AquaModel that affect fish growth and vary for each location are monthly average surface and bottom temperature and mixed layer depth, annual average inorganic nitrate concentration calculated based on temperature[36], bathymetry, and the 3D continuous ocean currents through the farm, which are estimated by the OCM. The OCM flow fields consisted of 6-hour averaged three-dimensional horizontal velocity and temperature for every day between April 2000 and

October 2001. Oceanographic and biogeochemical parameters besides those mentioned above were held constant throughout our study domain. These constant input parameters were based on the default values for the striped bass module within AquaModel and adjusted as needed to reflect local conditions of the Southern California Bight (Supplementary Data 2).

AquaModel is a computationally-intensive program requiring a high speed computer or long processing times on normal desktop computers (it takes several hours on a desktop computer to simulate one farm, as model time steps are hourly or less in most cases). Therefore, it was impractical for us to run the model for all 913 potential finfish sites (of the 1,134 sites developed for aquaculture, 913 fell within the 30-100 m depth limits assumed for finfish aquaculture) in our study domain. To overcome this issue, we estimated biomass production based on the environmental conditions of a subset of the potential sites, and then extrapolated the results to the remaining sites. In order to choose sites that represented the full breadth of environmental conditions experienced in the SCB, we grouped similar sites together using cluster analysis based on average summer sea surface temperature, surface current, bottom current, and depth. We restricted temperature data to summer months for two reasons: (1) summer is the time of highest growth rates and greater environmental impact, and (2) summer and winter temperatures were highly correlated but summer temperature showed more differentiation among spatial locations. Previous studies have reported a strong relationship between temperature, metabolism, and fish growth [37], thus in our analysis we weighted temperature more strongly (40%) than the other three 3 variables (20% each for surface current, bottom current, and depth). The cluster analysis used the squared Euclidian distance and between groups linkage, calculated using SPSS statistical software, resulting in the 913 sites being grouped into 36 clusters. We chose this level of clustering because it balanced capturing differences in environmental conditions among sites with the time required to run the model for each site. We then randomly selected two sites from each cluster group to run in AquaModel (except for one group that only contained a single site).

Using the output from the 71 model runs, we used ordinary least squares regression, implemented in EViews8 software, to estimate finfish aquaculture farm production for the remaining 842 sites. We randomly divided the two observations of each cluster into a training and test set. The training set observations, along with relevant environmental conditions (mean surface summer temperature, mean surface summer current, mean bottom summer temperature, mean bottom summer current, average annual inorganic nitrate concentration, mean winter mixed layer depth, and depth of the seafloor beneath the aquaculture farm) were inputted into a forward stepwise regression model algorithm in order of highest correlation coefficient to identify potential linear models for forecasting finfish biomass in a given site. We then performed cross validation with the test data set to evaluate the accuracy of each model and determine the best number of predictors; the predicted values were compared to the test set's actual values by calculating the test mean squared error, which was used as the primary performance indicator. We then selected the model with the lowest test mean squared error as our final model for predicting finfish biomass.

The final regression equation included mean summer surface current, mean summer surface temperature, mean winter layer depth, and inorganic nitrate as predictors for estimating finfish biomass production in the remaining 842 sites ($R^2 = 0.983$, SE = 80948.03; see Supplementary Data 2 for predictor coefficients).

We assumed that the grow-out period from stocked juveniles to harvestable adults was 1.5 years. As a result, the estimated biomass was divided by 1.5 in order to calculate the average

annual production of each developable site. We assumed that farms were not fallowed following harvest because our conservative farm design, water depths in which farms were sited, and current speeds in the Southern California Bight should preclude the need for fallowing. The model could be adjusted to include fallow years for contexts where that is the regulatory standard, which would result in far fewer sites being profitable for finfish aquaculture development.

The average annual production was then multiplied by a price of $8 per kg to determine average annual revenue (Supplementary Fig. 2c). This fixed price was based on price estimations of farmed striped bass[38].

Supplementary Note 8.

Finfish Aquaculture Cost Model: Costs for the finfish model were approximated based on projections for a previous industry proposal for southern California (Pers. Comm., D. Kent, Hubbs-SeaWorld Research Institute). The structure, parameters and assumptions of the fish cost model are consistent with the mussel cost model, with the following exceptions, many of which were determined by the aggregation level at which the data were shared with us (Supplementary Data 2; Supplementary Fig. 3c).

1. Starting costs were not calculated separately, but were instead incorporated into the annual operating expenses. This is due to the way that the fish cost data were aggregated, which integrated capital costs into yearly expenditures.

2. Since the proposed location of the farm for which we were basing our calculations was located in an area deeper than 50 m, we decreased the costs by 10% for all sites shallower than 50 m rather than adding costs for the deep sites (as was done with the mussel model).

3. All fish were harvested after 1.5 years, so there was no difference in the number of production cycles at each farm.

4. We estimated annual fuel costs directly as a function of distance from port (rather than estimating fuel consumption as we did with the mussel and kelp models). We calculated the annual cost of fuel at a rate of $15.00 per meter from port.

5. The production payroll costs were estimated to increase by $25.48 per meter from port. This was derived by multiplying estimated total labor costs by the percentage of employee time that would be taken up by transport to the site, and then dividing this by the distance from port.

Supplementary Note 9.

Halibut Fishery Biological Model: Aquaculture farms in the SCB could displace wild-capture fisheries. This conflict may be particularly strong (i.e., complete exclusion) for fisheries that use non-fixed gear (e.g., hook-and-line, trawls) to target fish that associate with nearshore soft-bottom habitat, as this type of activity would be prohibited in and around the farm because of concerns about gear entanglement. To represent this potential conflict, we modeled the *Paralichthys californicus,* or California halibut, fishery as it interacts with aquaculture development. *P. californicus* (hereafter referred to as halibut) is a flounder (Family *Pleuronectidae*) that associates with nearshore soft and mixed-sediment benthic habitat[39,40]. It is an important sport and commercial fishery species that is typically caught via hook-and-line, trawl, set gill net and trolling, and marketed as fresh fillet[41,42]. Commercial and recreational fishing occurs throughout much of the nearshore region of the SCB, except in marine protected

areas and other designated restriction zones (e.g., military, anchorage, navigation). Overall, the SCB halibut fishery is considered to be well-managed at a population level approximately equal to that associated with maximum sustainable yield (MSY)[41].

We developed an age-structured population growth model to simulate the growth, natural mortality, movement and recruitment of individual halibut. We then integrated the population model with a halibut fishery fleet model containing spatial, size limit and fishing effort level regulations. In the resulting coupled bioeconomic model, fishery profit is a function of revenue from harvest and market price, less the cost of fishing in relation to fishing effort, local stock density and site distance from port. Model initial conditions were calibrated relative to current estimated mean distribution of halibut biomass in the SCB. In order to represent fishery values important to both the commercial and recreational halibut fishery, we focused on fishery yield as the metric of annual value. Values to parameters described below are listed in Supplementary Data 2.

The model contains 4,518 1-km^2 nearshore sites in the SCB covering all soft and mixed-sediment benthic areas within halibut's preferred depth range (≤90 m)[42,43]. Experimental trawling in the SCB determined halibut abundance to be highest in shallow habitats and to decline to near zero at 90 m depth [43]. To approximate that pattern, we fit a set of functions (linear, logarithmic, exponential and power univariate functions) to the abundance-depth data and chose the function with the highest fit, the logarithmic function ($y=a*\text{Ln}(x) + b$, where x=depth and y=frequency of halibut occurrence; R^2=0.97; Supplementary Data 2). For each site in the model we multiplied the value of the function at the site's mean depth by the area of soft and mixed-sediment habitat in the site to generate a relative index of habitat availability, or $H_i=A_i*y_i$, where A_i is area of soft and mixed-sediment habitat in site i and y_i is the depth at the centroid of site i (see Supplementary Fig. 4a for a map of relative habitat indices).

The population model kept track of the number of post-recruit fish of each age class in each site and year, and their size (total length; cm) and weight (biomass; kg) in accordance with Von Bertalanffy growth[44] and allometric weight-at-length functions:

$$L_t = L_\infty\left(1-e^{-K(t-t_0)}\right), \text{ and} \qquad \text{(Sup Equ. 7)}$$

$$W = aL^b \quad , \qquad \text{(Sup Equ. 8)}$$

where t is age in years, L_∞ is the asymptotic fish length (i.e., mean maximum size), κ is fish intrinsic growth rate, and t_0 is theoretical fish age at size zero (Supplementary Data 2). The parameters a and b determine the multiplicative and exponential effect of fish length on biomass, respectively (Supplementary Data 2). Sexual maturity is reached for halibut at ~4 years old[16], and each year larvae are produced by reproductive-aged individuals at a rate proportional to their mass.

Halibut larval dispersal and site-to-site connectivity was estimated using a three-dimensional biophysical model, which consisted of the OCM (Supplementary Note 1) and a particle tracking model (PTM). The PTM was driven by six-hour averaged flow fields produced by the OCM that

moved particles forward in time using a fourth-order accurate Adams-Bashforth-Moulton predictor-corrector method[45,46]. The PTM was validated against observational data from drifter experiments[47].

Larval connectivity was quantified statistically using the Lagrangian probability density function (PDF) method[46,48] that estimates larval connectivity from a source site to a destination site by quantifying the probability of particle displacement over a specified time period. To incorporate the larval life history of halibut, the particles were tracked for 25 days, the PLD of halibut larvae, and released from May through December, the spawning period for halibut[41]. Additionally, halibut larvae have been documented to perform diel vertical migrations[40], which was programmed into the PTM. The particle release frequency was set at 12 hours to meet the criteria for robustness in PTMs[49]. Following the methods in Mitarai et al.[46], the coastline of the SCB was evenly divided into 135 coastal sites of approximately 75 km^2 each. Annual connectivity matrices were calculated between the coastal sites for each spawning season from 1996-2002 and then averaged over all years. The individual 1-km^2 halibut sites were assigned the connectivity values for the nearest coastal sites, producing a 6,425 site by 6,425 site dispersal kernel, a subset of which covers all 4,518 sites in the halibut fishery model.

Populations in the model are regulated by density dependent mortality occurring between larval settlement and recruitment[50]. Site-specific settler-recruit relationships are regulated by a Beverton-Holt function[51]. Each year the number of 'age one' fish recruiting in site i, N_{i1}, is dependent on number of settling larvae arriving into the site according to the OCM-generated dispersal kernal, S_i, and parameters α, representing the maximum recruit survival rate, and β_i, which regulates the maximum number of recruits possible in that site:

$$N_{i,1} = S_i R_i , \qquad \text{(Sup Equ. 9)}$$

where

$$R_i = \frac{\alpha}{1 + S_i \beta_i} \qquad \text{(Sup Equ. 10)}$$

and

$$\beta_i = \frac{\alpha}{R_{\max} H_i} , \qquad \text{(Sup Equ. 11)}$$

and R_{max} equals the maximum number of recruits possible per unit habitat, and H_i is the habitat availability index in the site.

Jointly, α and R_{max} set the strength of density dependence and affect fish population biomass and potential yield biomass levels in the whole system. Thus, we chose α and R_{max} to achieve empirically-estimated levels of density dependence and biomass levels of halibut in the SCB. We used the compensation ratio (CR) as our measure of density dependence[52,53], where CR describes the ratio between the maximum possible larval survival and larval survival in the unfished state, and is estimated to be CR=16 (corresponding with a steepness parameter h=0.8) for Family *Pleuronectidae*[41,54].

The commercial and recreational SCB halibut fishery, in the aggregate, is considered to be well-managed and approximately achieving maximum sustainable yield (MSY)[41]. Also, empirical records exist of annual commercial and recreational halibut landings in the SCB. Thus, we set R_{max} to generate, under MSY management conditions, a biomass yield equal to that measured empirically for the fishery in the SCB. In the model, MSY management was achieved by setting total allowable fishing effort (TAE), regulated across the entire study domain and distributed among fishable sites in accordance with a fishery fleet model (see below), to the level that maximized total sustainable yield. Empirical measurements of halibut biomass yield in the SCB were determined using the stock assessment by Maunder et al.[41]. Total abundance of halibut landed per year in the SCB by the recreational fishery (Table B1.6.1 in Maunder et al.[41] was multiplied by the average weight of a landed halibut by the recreational fishery (2.7686 kg; the mean of the PDF of recreational landings by fish weight; Fig. B2.8.2 in Maunder et al.[41]). These annual estimates of recreational fishery biomass yield were added to the annual biomass yield for the commercial fishery (Table B1.6.1 in Maunder et al.[41]). Total annual biomass yields were then averaged across 2004-2010 to estimate an average total halibut fishery yield in the SCB (177,779 kg per year). We focused our evaluation on the the most recent years in Maunder et al.[41], 2004-2010, because they are the closest to the present and these years represent the fishery when it is estimated to have been highly stable and at near-MSY conditions. Thus, in the bioeconomic model and given a total allowable fishing effort level generating MSY, R_{max} was set to return a total equilibrium annual yield equivalent to 177,779 kg per year.

Movement of post-recruit halibut among sites was simulated using a 2-D diffusion model parameterized with data from a mark-recapture study of halibut in and around the SCB. The 2-D diffusion model was modified to account for preferential movement in relation to habitat quality. For the diffusion model we used a normalized Gaussian function of a 2-D probability distribution that quantifies probability of movement from focal site $i=x$ to destination site $i=y$ in relation to the rate of diffusivity of the species, D, distance between the sites, $r_{x,y}$, and elapsed time, d. The probability of movement is assumed uniform in all horizontal directions; the analytical solution to this 2-D isotropic diffusion equation is known as Green's function[55,56]:

$$G_{x,y} = \frac{e^{\left(-r_{x,y}^2/4Dt\right)}}{4\pi Dd}, \qquad \text{(Sup Equ. 12)}$$

where d=365 days/year and $r_{x,y}$ is the distance between the centroids of sites $i=x$ and $i=y$. For calculating site fidelity within a site, $y=x$. We assumed the system was closed with respect to halibut movement, and thus standardized Sup Equ. 12 so that the probability of movement from each focal site to all sites in the domain sums to one:

$$G_{x,y}^S = \frac{G_{x,y}}{\sum_{y=1}^{Y} G_{x,y}} \qquad \text{(Sup Equ. 13)}$$

When exhibiting directed movement, animals generally have higher movement rates toward sites of higher habitat quality (and/or will stay in their current site if it is of higher habitat quality than nearby sites). Following Cheung et al.[57], we modeled such behavior by incorporating a hyperbolic function into the calculation of halibut movement:

$$G_{x,y}^{S,k} = \frac{kG_{x,y}^{S}}{K + H_{i=y} / H_{i=x}}, \qquad \text{(Sup Equ. 14)}$$

where k is a scaling factor representing the sensitivity of the calculated movement rate to changes in habitat quality[58], which is indicated in the denominator by the ratio of habitat quality relative to the focal site. Small values of k (e.g., k=0.1) result in high sensitivity to the habitat ratio, while large values (e.g., k=10) render adult movement rate insensitive to the habitat ratio. We used an intermediate value, k=2, used previously in the literature for marine fishery species[57]. Finally, to maintain a closed system of adult movement we rescaled the solution to obtain the realized rate of movement, $M_{i,i}^{a}$, that we used in the population model:

$$M_{i=x,i=y}^{a} = \frac{G_{x,y}^{S,k}}{\sum_{y=1}^{Y} G_{x,y}^{S,k}}, \qquad \text{(Sup Equ. 15)}$$

We used the exponential survival function for calculating post-recruit halibut mortality in relation to instantaneous natural mortality rate, M, and fishing mortality rate, F_i, which is equal to site-specific fishing effort multiplied by a catchability coefficient, $E_i q$, and applied only to legally-harvestable halibut age classes. Without loss of generality, we set q equal to 1. Legal-to-harvest age limit was set to 5 years old, corresponding with the legal-to-harvest size limit of 22 inches in total length for the SCB halibut fishery and the conversion from size to age determined by Sup Equ. 7-8. Instantaneous natural mortality rate was set to M=0.25, the average of female and male rates used in the stock assessment of the species in southern California by Maunder et al.[41]. Fishing effort (E_i) was determined in relation to TAE and the fleet model.

In site i, the number of fish of age $j+1$ at the end of the year, $N_{i,j+1\_end}$, is a function of the natural and fishing mortality rates in the site and of the population of fish age j at the beginning of the year:

$$N_{i,j+1\_end} = N_{i,j\_beginning}\, e^{-(F_i + M)} \qquad \text{(Sup Equ. 16)}$$

where F_i equals zero for all age classes j that are not legal to harvest.

For each legally harvestable age class, the site-specific proportional biomass loss due to mortality is equal to $1 - e^{-(F_i+M)}$. Of this mortality loss, the amount attributable to the fishery (as opposed to natural mortality) is proportional to the relative rate of fishing versus natural

mortality in that site, $F_i/(F_i + M)$. Thus, in each year fishery yield per site per fishable age class j is:

$$Y_{i,j} = N_{i,j\_beginning}(1 - e^{-(F_i+M)})(F_i / F_i + M) \quad \text{(Sup Equ. 17)}$$

Halibut also have a maximum age of ~27 years[41], although very few individuals reach it. At the maximum age, natural mortality is 100%.

Given the above parameter values, Supplementary Fig. 4b illustrates the model estimate of equilibrium virgin (unfished) spawning stock biomass of halibut across the study domain with F_i=0. At MSY our model estimated spawning stock biomass to be 24.24% of virgin spawning stock biomass; this has been considered a reasonable reference point of sustainable halibut fisheries management[41] and thus a indication that our model is representing the status of the fishery resonably well.

Supplementary Note 10.

Halibut Fishery Economics and Fleet Model: Commercial and recreational halibut fishing is restricted by numerous conservation, military, navigation and other regulated zones across the study domain. Thus in our model we restricted positive fishing effort from all sites within those areas (Fig. 1a).

In order to represent fishery values important to both the commercial and recreational halibut fishery, we focused on fishery yield as the metric of annual value (for calculating net present value and annuity), and, given that the fishery is considered to be well-managed and approximately achieving maximum sustainable yield (MSY)[41], in our model we set total allowable fishing effort (TAE) to the level that generated MSY. However, it is also of interest to know the potential economic value of the fishery in terms of profit. Fishery profit is a function of total revenue from harvest (TR) less the total cost of fishing (TC):

$$\pi = \sum_i (TR_i - TC_i), \quad \text{(Sup Equ. 18)}$$

Total revenue is a function of market price and yield,

$$TR_i = pY_i \quad \text{(Sup Equ. 19)}$$

We set price to p=\$10.67 per kg (\$4.84 per lb), equal to the ex-vessel dollar value divided by biomass landings of commercial halibut in southern California by year, averaged across 2004-2011, the years when the halibut stock assessment confirmed the catch and biomass to be relatively stable[41]. Linear and exponential regression of annual price in relation to biomass landings did not reveal a significant relationship that would be indicative of the presence of an inelastic demand curve (i.e., reduction in price with increased supply). We also used regression analysis to test for a negative relationship between annual price of halibut and biomass landings of California white seabass (white weakfish; *Atractoscion nobilis*)[41], which is a high-value seafood species with a mild, firm and flaky white meat that is marketed as fish fillet and is similar to the striped bass, *Morone saxatilis*, represented by our aquaculture finfish model. This

regression analysis also returned non-significant results. Consequently, we assumed halibut price to be constant in our model analysis.

In our model, cost of fishing per unit area in each site was initially calculated in relation to the change in stock density in the site over the fishing period due to fishing (and natural) mortality.

$$TC_i^a = \int_{s_i=\left(N_{i,end}^{legal}/H_i\right)}^{s_i=\left(N_{i,beginning}^{legal}/H_i\right)} c(s_i)\partial s \qquad \text{(Sup Equ. 20)}$$

where s_i is the biomass density of legal-to-harvest fish in site *i.* Marginal cost, $c(s_i)$, is a decreasing function of resource stock density in the site (i.e., $c'(s_i)<0$; i.e., higher fish stock density reduces per-unit harvest cost). We modelled marginal cost of fishing to be inversely proportional to local fish density, $c(s_i) = \theta/s_i$, where parameter θ determines the stock density below which marginal cost equals price and thus fishing is expected to naturally cease (higher values of θ represent species that are intrinsically more expensive to harvest)[59,60]. Fishing cost and its effect on fishing behavior are difficult to quantify[61], but in general it is sometimes assumed that marginal cost equals price when the legal-to-harvest fish stock is reduced to 10% of its virgin carrying capacity, and at this level represents the "break even" stock density below which it is unprofitable to fish and the fishing naturally ceases[16,60]. Ten percent of virgin stock density also is commonly considered to indicate collapse of a stock and its associated fishery[62]. Consequently, we set θ in order to produce a marginal cost equal to price at a fish stock density equal to 10% of the mean virgin legal-to-harvest stock density across the study domain. Cost was then converted from per unit area to per unit site, $TC_i^p = TC_i^a H_i$.

Cost of fishing also is expected to be a function of travel distance to the fishing ground[63]. To include this factor, cost of fishing in a site was modulated in relation to its level of isolation,

$$TC_i^\gamma = TC_i^p\left(1+\gamma D_{i-port}\right), \qquad \text{(Sup Equ. 21)}$$

Where γ is a scaling parameter and D_{i-port} is the distance in meters from site *i* to its nearest port. Finally, cost is incurred by the fishery to the extent that the observed reduction in stock density is due to fishing effort, as opposed to natural mortality. Consequently, the cost in a site to the fishery is a function of the proportion of morality in the site due to fishing versus natural mortality,

$$TC_i = TC_i^\gamma\left(\frac{F_i}{F_i+M}\right) \qquad \text{(Sup Equ. 22)}$$

Empirical data on the spatial distribution of halibut landings were used to set the travel cost scaling parameter γ. Empirical measurements of commercial and recreational halibut fishery yield in the SCB were determined using Pacific Coast Fishery Geographic Information Systems fisheries landings data ("PacCoastFisheryGIS" data) generated under the California Department of Fish and Wildlife Statewide Marine Protected Area Management Project. The spatial

resolution of the data corresponds with the California Department of Fish and Wildlife 1' (~10 km) fishing blocks, of which there are 87 in the SCB in our model domain. Overall, the empirical data indicates halibut landings to be concentrated along the mainland and near ports (Supplementary Fig. 5a), supporting the assumption that travel cost influences the spatial distribution of fishing effort[63].

Using the PacCoastFisheryGIS data, we "tuned" γ in order to minimize the sum of squared error (SSE) between the empirical data and our model estimate of the proportion of total biomass landings in the study domain from each reporting block. The tuning procedure generated minimum SSE=0.017113, and linear correlation between the empirical and model values of R^2=0.62. The resulting model estimate of the spatial distribution of landings is shown in Supplementary Fig. 5b. Given the tuned value of γ (6.0708e-05), travel costs increased total annual fishing cost in a site by a factor of ~0-4 along the mainland and ~2-7 at the Channel Islands, depending on their specific distances from the nearest port.

In the California halibut fishery and other limited-entry fisheries, the spatial pattern of fishing effort (e.g., fishermen, boats) among sites is expected to be a function of the relative value of the sites; this pattern can be estimated using a fleet model[16,64,65]. Consequently, we determined the spatial allocation of the total allowable effort (TAE) by the fishery using an ideal free distribution[66] fleet model, such that average profits (profit per unit effort) were equal among all fishable sites (i.e., those that contain halibut and are non-MPA, non-military, aquaculture, etc. sites; Supplementary Figs. 1 and 4a). Note that this behavioral model of fleet dynamics, while potentially representative of the actual relationship between vessel and resource distributions in fisheries[67,68], is not necessarily expected to produce the optimal pattern of spatial effort distribution (e.g., by a sole owner or fishery cooperative) that maximizes the total value of the fishery[69]. MSY management was achieved by setting total allowable fishing effort (TAE), regulated across the entire study domain and distributed among fishable sites in accordance with the fleet model, to the level that maximized total equilibrium yield. As explained above, parameter R_{max} was set to achieve a MSY matching that estimated empirically (177,779 kg per year).

Equilibrium MSY management was used as the initial conditions in the model when evaluating effects of aquaculture development on the halibut fishery. Given an aquaculture development plan, sites with aquaculture were closed to halibut fishing immediately (i.e., in year 1) and continuing through year 10 (the end of the evaluation time horizon), and Net Present Value of the halibut fishery was calculated in response to this closure (Eq. 1-2).

In order to make the halibut bioeconomic model compatible with the tradeoff model framework, which required static models for our optimization approach, we assumed fisheries value in each of the 10 years following aquaculture development to be represented by the yield values generated under MSY conditions, but with zero yield in the new closures due to the aquaculture farms. This simplification excludes the ecological dynamics of larval dispersal and fish spillover, and socioeconomic dynamics affecting changes in the spatial distribution of fishing effort among fishable sites, that are present in our dynamic model and could occur in response to new closures from aquaculture farms. However, our focus on static model values is not expected to generate significantly different results compared with if the fully dynamic model were incorporated into the tradeoff analysis, for two reasons. First, the halibut fishery already is managed at MSY, and thus there are unlikely to be dramatic ecological or fleet behavior changes from closing some sites to fishing. Second, the time horizon is short (10 years), limiting the

development of a long-term dynamic response to a change in management decision; i.e., the short-term dynamic response is not much different than the static response estimate. Both of these reasons are supported by Brown et al.[70], which specifically compared dynamic and static models for conducting MSP. Nonetheless, in order to provide the most accurate estimates possible, once an MSP solution (i.e., aquaculture farm design on the 7-D efficiency frontier) had been identified by the tradeoff model, we then evaluated its effect on the halibut fishery using the fully dynamic bioeconomic model. In that case, sites with aquaculture were closed to halibut fishing, the bioeconomic halibut fishery model was simulated forward in time 10 years, and yield in each year was recorded. We also used the fully dynamic bioeconomic halibut fisheries model for evaluating effects of the plans derived under conventional planning. Consequently, we estimated and reported the actual effect on the halibut fishery of all of the aquaculture farm designs presented in this study based on the fully dynamic halibut model described above.

Supplementary Note 11.

Viewshed Model: New development can change the aesthetic qualities of a place. The potential impact of aquaculture development or other emerging uses on views and scenic values is a frequent concern expressed by stakeholders[71,72]. Though scant evidence exists that offshore aquaculture development decreases coastal real estate values[73], community members may still oppose aquaculture development because they are concerned about changes in scenic values and impacts on tourism, recreation, and property values. For these reasons, other regions have modeled and analyzed the visual impact of new aquaculture development on surrounding areas[73,74]. We developed a modeling approach that assesses how many people's views could be affected by placement of a farm in each site, incorporating both residential views and recreational views from state parks and beaches.

We ran the ArcGIS 10.2 Viewshed tool to identify all locations on land from which a given developable site would be visible within a defined radius of visibility. Based on previous work and our assumptions about farm design, we assumed that mussel and kelp farms would not be visible beyond a 3-km radius[75], while finfish farms would be visible up to 8 km away[73]. We assumed the height of a viewer on land to be 1.7 m tall (the average height of adult men and women in the United States) and infrastructure of all farm types would be primarily horizontal structures on the water plane (including linear patterns of buoys for kelp and mussels, net pens for fish, and navigational lighting for both), extending vertically less than 1 m. Inputs to the model were the locations of the centers of each developable site (a shapefile of points) and a high resolution (90-m) raster digital elevation model of land (DEM; Supplementary Data 1). Data were not available to incorporate trees or buildings into the DEM, though we acknowledge that both could affect actual visibility from a given location (i.e., blocking views or allowing farms to be viewed further away from an upper story of a building). We used an earth curvature correction factor and a refractivity coefficient of 0.13, the default settings for the Viewshed tool. This part of the analysis produced rasters of which sites on land can view each farm. We then determined how many people's views would be impacted given the distributions of residential populations and visitors to state parks and beaches within impacted sites on land, for each possible aquaculture site.

For residential views, we summed the number of people living in impacted view areas on land using a 30-m grid of human population density supplied by the Natural Capital Project[76]. For scenic and recreational views, we assessed which farms would be visible from state parks and beaches and calculated the number of park or beach visitors who would potentially be affected

by that farm location, based on total annual visitor attendance data (day use and campers) from the 2011/12 Fiscal Year[77]. Because there are not data on where visitors spend time within a state park or beach, we assumed that a view of the farm from any part of the park or beach would affect all visitors. Also, we recognize that there are other important locations for scenic recreational viewing beyond state parks and beaches (e.g., county parks, coastal roads and highways), but we were not able to obtain consistent use data across our study domain for these other areas and so just focused our analysis on state parks and beaches. Lastly, it is important to note that not all state parks and beaches collect and/or report visitation data, so this dataset is far from complete, and should be treated as a demonstration of what is possible rather than a definitive estimate for where impacts would be highest. For an actual permitting or planning exercise, it would be important to fill gaps in the scenic and recreational dataset.

The outputs of the viewshed analysis were rasters of which sites on land can view each farm, summed residential population within the viewshed of each farm, state parks and beaches within the viewshed of each farm, summed annual visitors to state parks and beaches within view of each farm, and the total number of people whose views would be affected by each farm (summed across residential and park/beach visitors). For the calculation of value for this sector used in the tradeoff analysis, we combined residential and recreational views into a single metric representing the number of people whose views could be affected by the development of each site (Supplementary Fig. 6a,b). The weighting of residential versus recreational viewshed impacts could be adjusted to reflect the relative importance of the two components to stakeholders or decision-makers in a particular planning context. We chose to sum the number of people because there was not information for southern California upon which to judge the relative value stakeholders place on uninterrupted residential versus recreational views.

Supplementary Note 12.

Benthic Environmental Health Model: Offshore aquaculture can generate negative environmental impacts, particularly with regards to pollution, and limiting negative impacts is typically a key focus in planning for future aquaculture development[5,78,79]. The magnitude of these effects is generally heavily influenced by operational characteristics, such as species farmed, stocking density, and feeding strategy, but also by farm location[79] and thus spatial planning should take into account spatial variability in possible environmental impacts. Specifically, the physical and chemical characteristics of the surrounding environment, such as background nutrient levels, currents, and depth, are important in determining the fate and impact of any pollutants released from the farm[80,81]. Both fed and unfed aquaculture operations release particulate organic matter (such as feces, or uneaten feed in the case of fed aquaculture) that can settle to the seafloor where it can lead to eutrophication and local oxygen depletion in and near the benthos[82,83]. The community level effects of increased nutrients and decreased oxygen on the benthos can vary significantly based on the level of impact and background conditions; indeed, low levels of nutrient enrichment can have a minor effect and may even increase benthic biodiversity and biomass[84,85]. However, for the purpose of this analysis we take a precautionary approach that assumes that any changes to the benthic environment are not desirable. Generally deeper water and faster currents result in more diffusion of organic material, which will minimize any adverse effects, but also create a larger footprint of benthic areas potentially affected by the farm[80,81]. While shellfish operations have been shown to have benthic impacts in shallow sheltered areas, field studies of offshore shellfish operations have shown that there is unlikely to be any benthic impact in the deeper open ocean environments that are generally

typical of offshore aquaculture operations[86]. We therefore focused on impacts to the benthos only from finfish aquaculture, since this impact often is listed as a primary concern by the aquaculture industry and public stakeholder groups[5,79].

Benthic impacts were estimated using AquaModel (Supplementary Note 7). Specifically, we used the model to estimate the total organic carbon (TOC) flux to the seafloor as a proxy for the risk of a given farm producing hypoxic or anoxic conditions on the seafloor near the farm. AquaModel simulates both the flux of particles containing organic carbon and the resuspension and re-distribution of these particles as they are assimilated by the benthic food web. We focus on organic carbon flux because organic carbon is the source of the potential problems to sediments (such as shifts to anaerobic bacterial dominance when the rate of deposition exceeds the aerobic assimilative capacity). Furthermore, the effect of additional organic carbon on the biogeochemical characteristics of the sediment and the microbenthic infauna are well understood [87], and these measures are the basis for all aquaculture sediment effect models. We calculated the average TOC flux to the seafloor (g per m^2 per day) at each aquaculture site to provide a relative measure of potential organic enrichment. This measure does not account for resuspension rates, so is a conservative index of potential effect and allows us to identify sites that may be at higher risk to experience benthic impacts. The model runs used to estimate TOC flux were the same as described in Supplementary Note 7 to estimate biomass production. The same method, including cluster analysis and stepwise regression, was used to extrapolate from the 71 sites that were run in AquaModel to estimate TOC rate for the 842 potential finfish sites that were not run in AquaModel (Supplementary Fig. 6c). The coefficients in the regression equation can be found in Supplementary Data 2 ($R^2 = 0.83$, SE = 0.055).

Supplementary Note 13.

Disease Risk Model: Risk of disease outbreak often is used as a justification for opposition to aquaculture development[79]. Two primary types of infectious agents are often identified as vectors for aquaculture disease, viruses and bacteria[88,89]. While the widespread use of antibiotics and vaccines have been very effective against bacterial pathogens, viral infection remains a significant problem for the aquaculture industry[90]. Viral disease risk appears to be highest for finfish farms (relative to shellfish and algae), due to the use of feed made from the carcasses of other fish species, which greatly increases the risk of exposure to novel pathogens[88,89]. Although the risk of viral disease outbreaks is strongly influenced by husbandry practices (stocking density, feed characteristics, etc.), there is also a spatial planning dimension to disease risk. Specifically, viruses could potentially travel between farms via pelagic dispersal, meaning farm design and farm density in relation to ocean currents could influence the risk of disease transmission and system-wide outbreak. We therefore focused on modeling oceanographic connectivity among finfish farms as an indicator of risk of viral disease outbreaks in relation to alternative spatial plans of finfish aquaculture development.

Marine viruses have life stages in which they are inside their host (e.g., a fish) and dispersing between hosts in the water[91]. Because finfish are contained within the farm pens, we focused on the dispersive stage of the virus in connecting farms and potentially spreading disease. To do this, we utilized the three-dimensional ocean circulation model (Supplementary Note 1). When outside of their hosts, viruses degrade rapidly within a matter of hours due to UV radiation[92]. Thus, in the OCM we simulated viruses with a dispersal period (akin to a “pelagic larval duration”) of one day, in order to estimate the distance a single virus could travel within the study domain before being destroyed by UV exposure[49]. Using the simulation outputs we

generated a probability density function (PDF), or dispersal kernel, for each developable finfish site given the fixed spatial constraints (see Methods in main paper). The dispersal kernel for disease transport was estimated using the same methods described for halibut larval dispersal (Supplementary Note 9), except that in the particle tracking models the particles were released year-round and only from sites that may be developed for finfish aquaculture (i.e., of positive NPV value to that sector), and they dispersed passively (i.e., no vertical migration or other behavior) for 24 hours.

We applied network analysis to the viral dispersal kernel to estimate relative risk of a system-wide disease outbreak of a given finfish farm plan. The goal of the network analysis was to calculate the degree of centrality of each potential fish farm site to all other potential fish farm sites, then use this information to penalize farm plans that develop highly central "hub" sites most likely to support disease transmission among farms throughout the study area. We did this by evaluating the eigenvector centrality for each potential finfish farm site in a proposed spatial plan. Eigenvector centrality is often cited as a primary way to identify disease hubs in weighted networks[93,94]. Unlike many other centrality metrics, eigenvector centrality takes into account the complete topology of the entire network, instead of just assessing the strength of direct connections, meaning that the centrality of a single node is dependent on both direct and indirect connections between nodes (in our case, farms). To calculate eigenvector centrality for each site we first estimated maximum potential disease risk by considering the scenario where all potential finfish aquaculture farms are developed. Using the dispersal kernel as the adjacency matrix for the disease network, we calculated the eigenvector centrality of each developable site using the equation from [95]:

$$Ax_i = \lambda x_i \qquad \text{(Sup Equ. 23)}$$

where x is the eigenvector of a given site i in the disease adjacency matrix, A, with an eigenvalue, λ. As explained by the Perron–Frobenius theorem, λ is the largest eigenvalue and x the corresponding eigenvector[96] for a given site. Thus, the value λ represents the degree of centrality for a given site, which we used to represent the disease response to finfish aquaculture at that site. For a given spatial plan of aquaculture development, total risk of disease spread of the entire plan was calculated as the sum of the eigenvector centrality values of the developed sites (Supplementary Fig. 6d).

Supplementary Table 1: Steps for implementing the full MSP analytical model.

Step	Significance	Case study description
• Specify study domain<br>• Identify emerging sectors and key existing sectors of concern in relation to emerging sector development	• Delineates the spatial planning problem<br>• Acknowledges environmental impacts and socioeconomic conflicts that may be generated by emerging sectors	• Study domain: Southern California Bight<br>• Emerging sectors: mussel, finfish and kelp aquaculture<br>• Impacts and existing sectors: halibut fishery, benthic health, viewshed quality, risk of disease outbreak
• Identify and delineate potential development locations for emerging sectors	• Constrains spatial planning problem to feasible sites for development	• Aquaculture development restricted from fixed sites: military areas, shipping lanes, protected areas, rocky substrate, minimum and maximum depth zones, and areas around sewage outfalls and major river mouths<br>• Identified 1,061 feasible sites for at least one type of aquaculture
• Generate spatial models of the existing and emerging sectors and their interactions	• Explicitly characterize interactions between existing and emerging sectors	• Spatially-explicit biological and socio-economic models of the three emerging and four existing sectors, modelled across all feasible sites
• Apply all potential policy options to the sector models across all feasible sites	• Quantifies site-specific sector responses to the alternative policy options<br>• Sector responses can represent values or impacts, and can be in different units	• Model response of each sector to each of four policy options at each site: no development, or development of mussel, finfish or kelp aquaculture<br>• Aquaculture and halibut fishery sector responses represent value (in economic annuity)<br>• Benthic health, viewshed quality and risk of disease outbreak sector response represent impact (in various units)
• Convert impacts into values<br>• Estimate the value to each sector for each policy option at each site	• Aligns sector valuations, such that, for all sectors, higher numbers are more beneficial to the sector	• For benthic health, viewshed quality and risk of disease outbreak sectors, value for a policy option at a site calculated as the maximum response by the sector across all sites and policy options minus the sector's modelled response to the policy option at the site

		• For aquaculture and halibut fishery sectors, value is equal to the sector's modelled response
• Transform all sector values to a common, unitless scale (i.e., where the value equals the proportional contribution of each policy option at each site to each sector's total potential value)	• Transforms sector values into comparable units	• For each sector, its scaled value is the value of the sector at a given site, under a given policy option, divided by the maximum total potential value (i.e., the summed value if the ideal policy option for that sector was selected at every site)
• Apply weighting factors to each sector's scaled values	• Accounts for different relative socio-political preferences for each sector<br>• Different combinations of preference weights can be used to evaluate different priorities, or when priorities are uncertain or unspecified	• All combinations of six weights $\{\alpha_n = 0, 0.2, 0.4, \ldots 1\}$ were applied across the seven sectors, generating $6^7 = 279{,}936$ results indicating the weighted value to a sector at a site from implementing one of the policy options
• For a particular set of preference weights, generate a marine spatial plan solution by selecting the policy option at each site that maximizes the sum of weighted sector values<br>• Calculate the domain-wide outcome of that plan for each sector	• Indicates an optimal spatial plan, and associated sector impact and/or value, in relation to specified priorities across sectors (i.e., preference weights)	• The marine spatial planning solution illustrates the optimal policy option (i.e., aquaculture development option) for each of the 1,061 sites, where, for each site the selected option best maximizes the sum of the seven weighted sector values<br>• Outcome indicates how the plan will affect each sector in terms of its response and/or proportional change in impact or value
• Replicate the above step for each set of sector-specific weights	• Identifies the set of spatial plans that delineate the efficiency frontier of marine spatial plan solutions that optimally balance the tradeoff in values and impacts among the interacting sectors	• A seven-dimensional, 279,936-point efficiency frontier of marine spatial plan solutions of optimal aquaculture development in relation to the values of the three aquaculture and four existing sectors and their interactions

Supplementary Figures

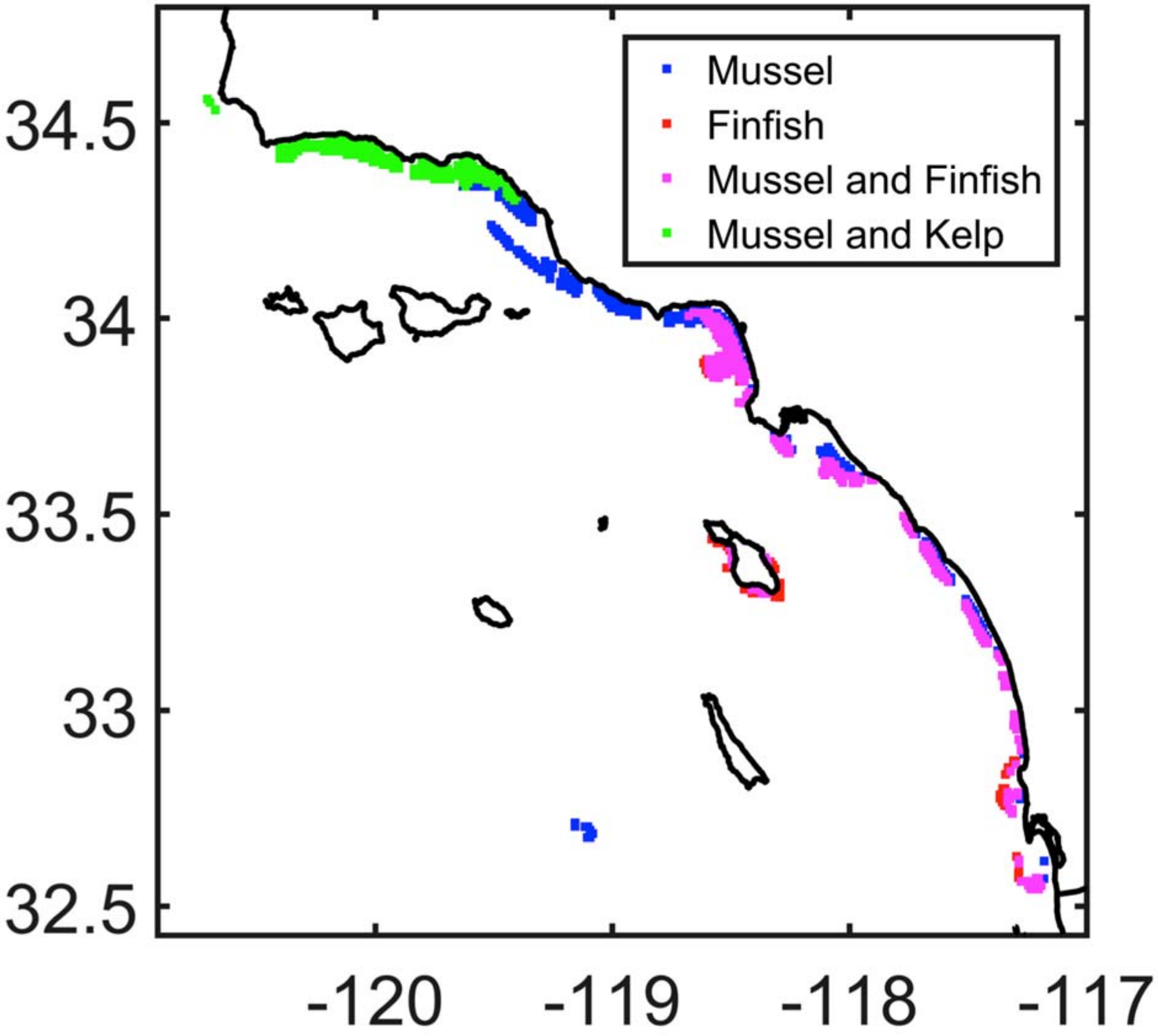

Supplementary Figure 1: Development domains of the three forms of aquaculture, given the imposed logistical, sociopolitical, and economic constraints.

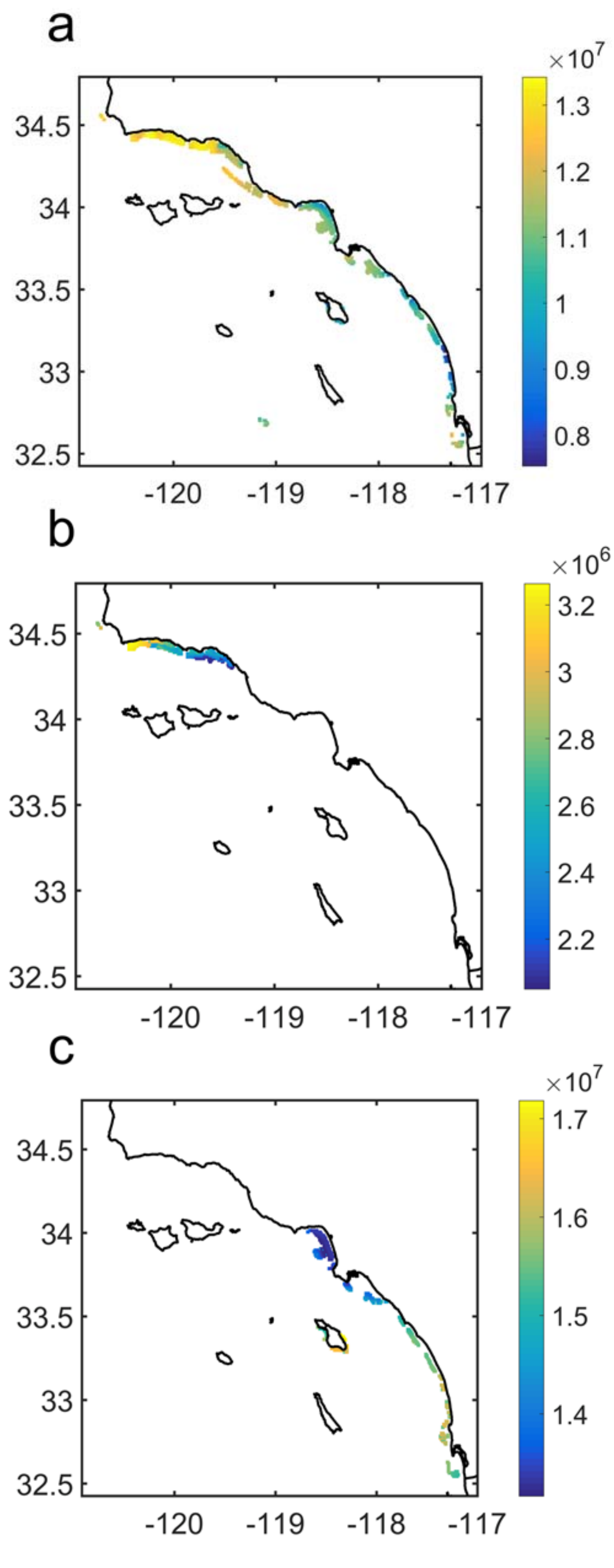

Supplementary Figure 2: Modelled annual revenue of (a) mussel, (b) kelp, and (c) finfish aquaculture for each site, in US dollars.

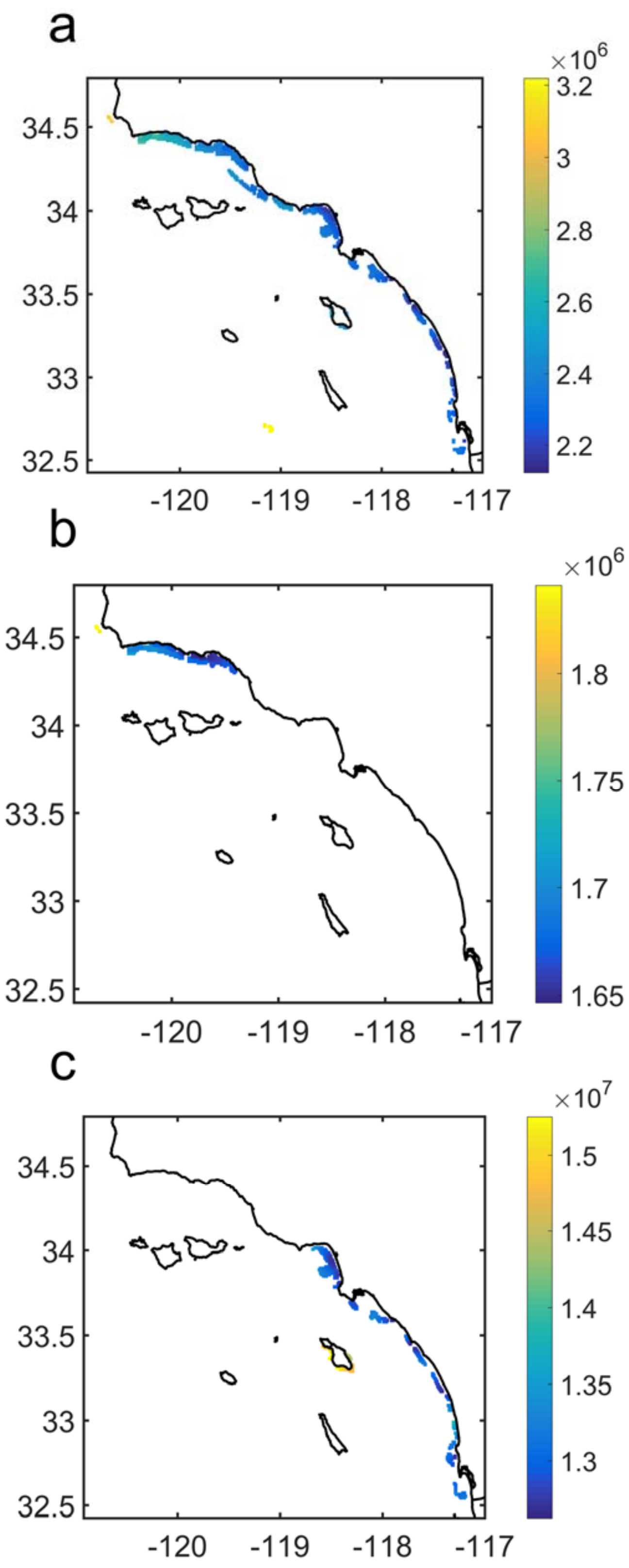

Supplementary Figure 3: Potential annual costs of (a) mussel, (b) kelp, and (c) finfish aquaculture for each site, in US dollars.

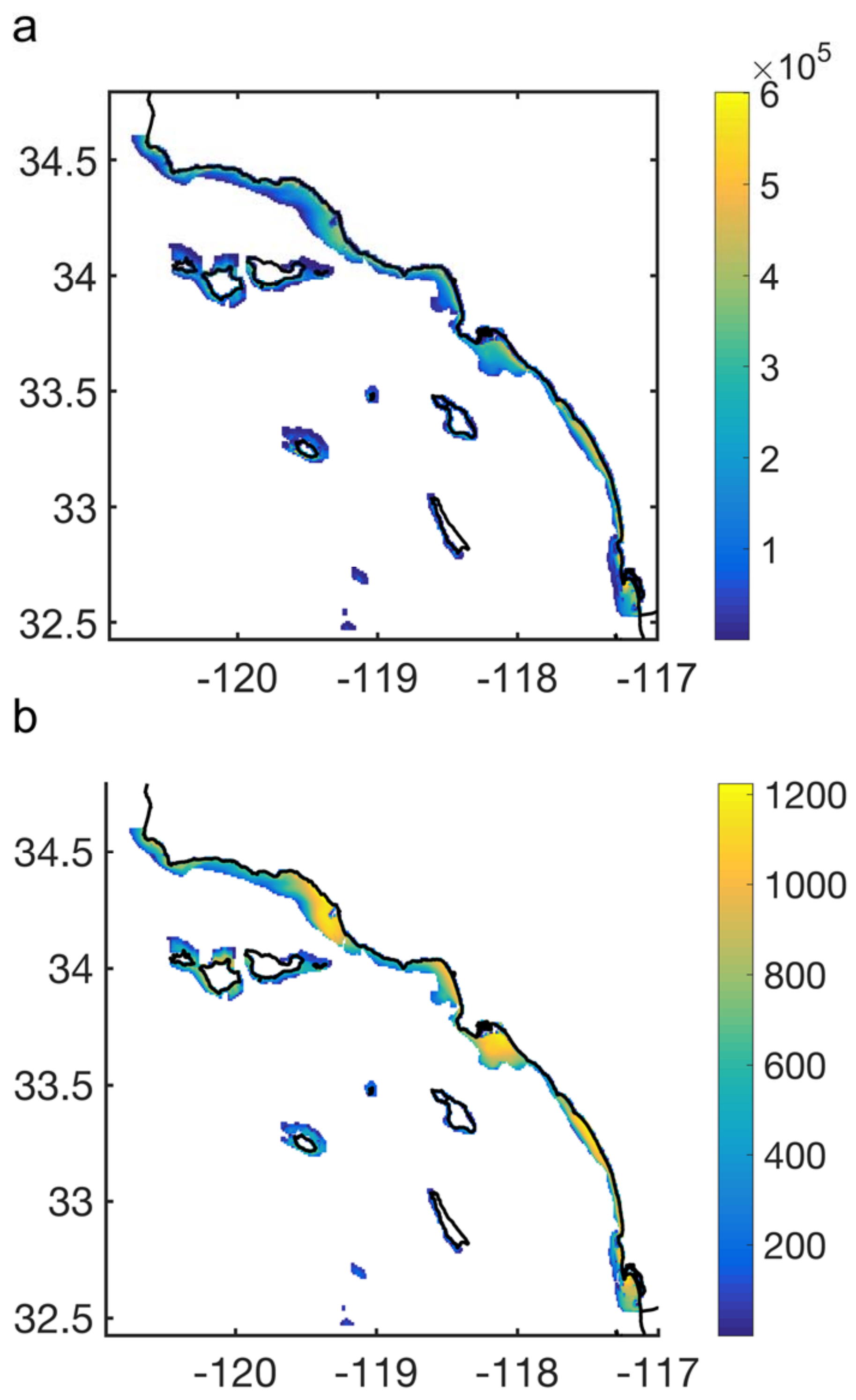

Supplementary Figure 4: (a) Halibut habitat index, used for characterizing relative availability of suitable habitat in each site. The index was calculated for each site as the logarithmic function derived from the Haugen[43] data and evaluated at the site's depth, multiplied by the area soft and mixed-sediment habitat in the site. (b) Model estimate of halibut spawning stock biomass density (kg per km^2) in a world without fishing.

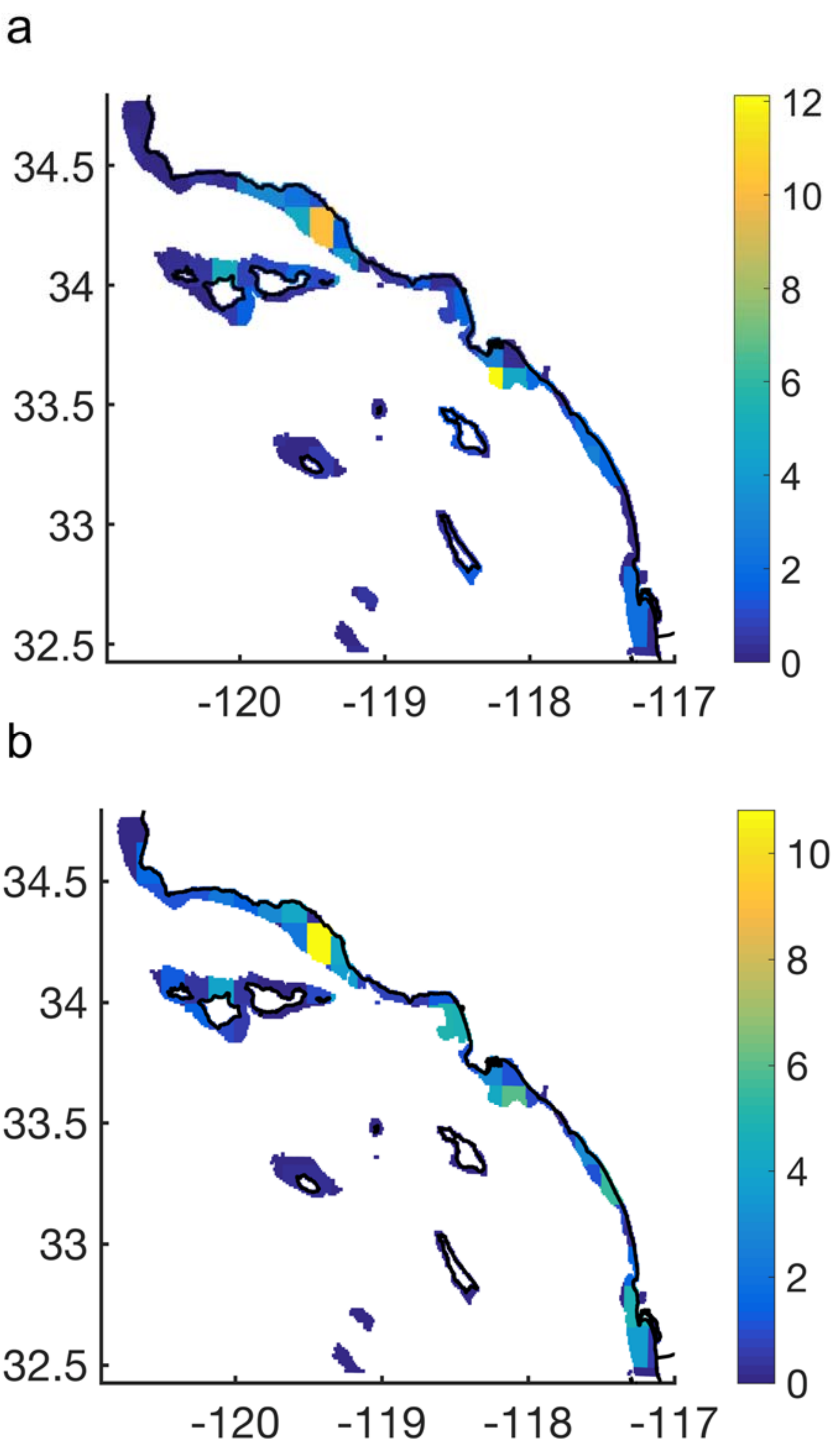

Supplementary Figure 5: (a) Empirical data of the proportion of total halibut fishery landings in each California Department of Fish and Wildlife reporting block. (b) Model estimate of halibut fishery landings (kg per km^2).

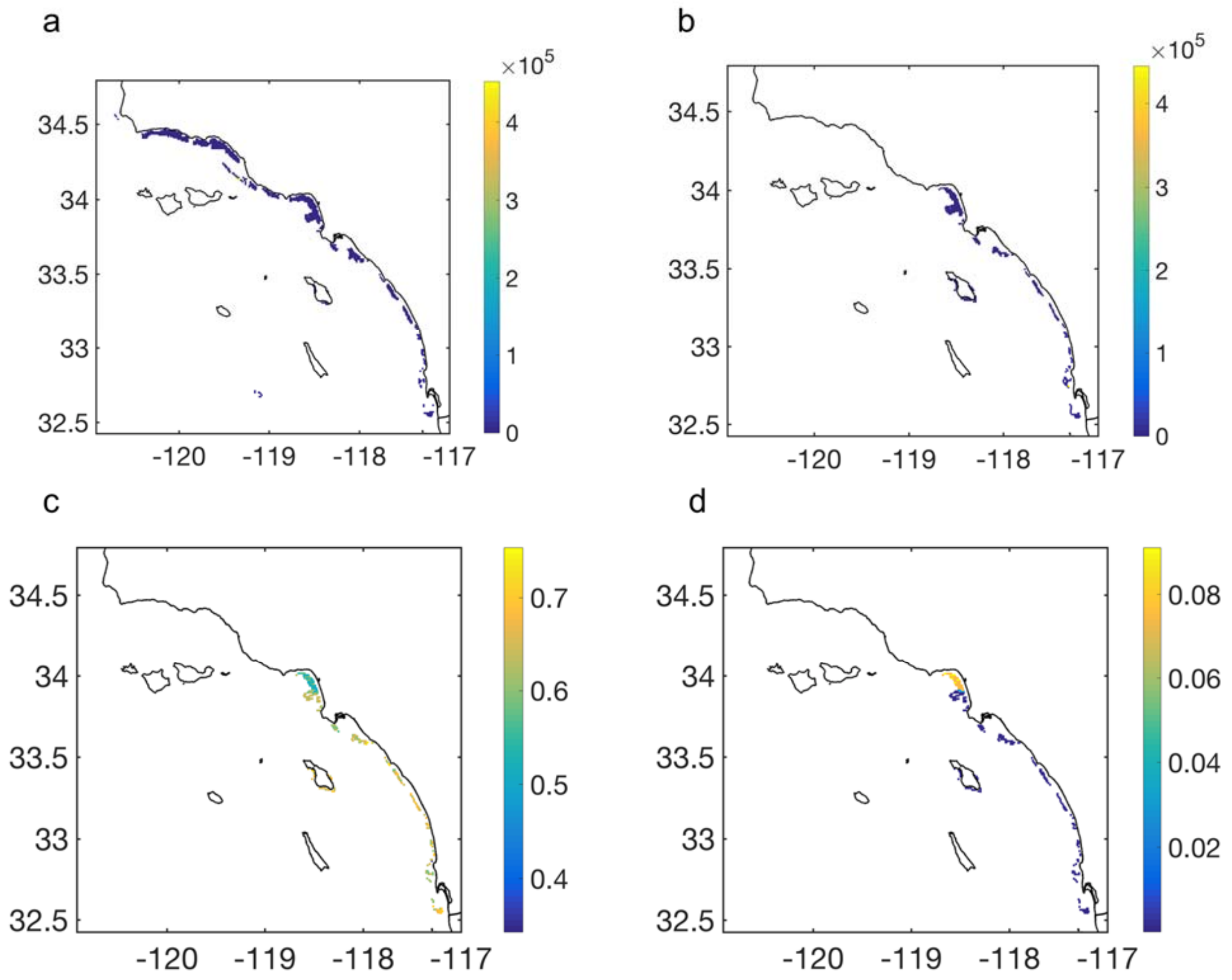

Supplementary Figure 6: Coastal viewshed impact (number of impacted people by a farm) of mussel and kelp farms (a) and finfish farms (b) at each site. (c) Benthic impact (TOC flux) for each developable finish site. (d) Potential disease risk (in terms of eigenvector centrality) for each developable finfish site.

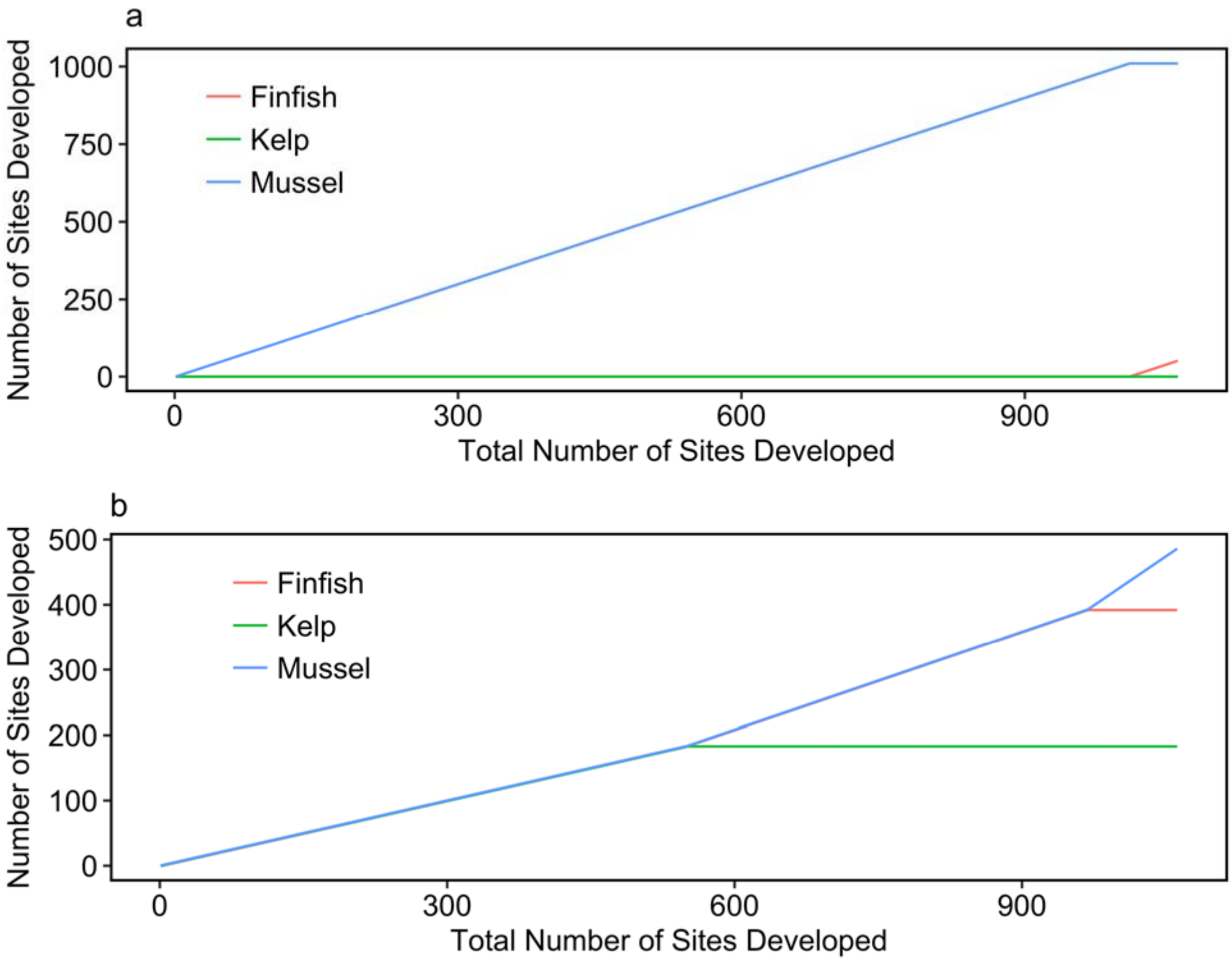

Supplementary Figure 7: Number of sites developed by each aquaculture type, given unconstrained (a) and constrained (b) approaches to conventional planning.

Supplementary References

1 Shchepetkin, A. F. & McWilliams, J. C. The regional oceanic modeling system (ROMS): a split-explicit, free-surface, topography-following-coordinate oceanic model. *Ocean Modelling* **9**, 347-404 (2005).

2 Dong, C. & McWilliams, J. C. A numerical study of island wakes in the Southern California Bight. *Continental Shelf Research* **27**, 1233-1248 (2007).

3 Dong, C., Idica, E. Y. & McWilliams, J. C. Circulation and multiple-scale variability in the Southern California Bight. *Progress in Oceanography* **82**, 168-190 (2009).

4 Wang, C., Deser, C., Yu, J.-Y., DiNezio, P. & Clement, A. El Nino and southern oscillation (ENSO): a review. *Coral Reefs of the Eastern Pacific*, 3-19 (2012).

5 Department of Fish and Wildlife. Marine Region Information Leaflet Regulations Governing Marine Aquaculture. (The Natural Resources Agency of California, Available at: https://nrm.dfg.ca.gov/FileHandler.ashx?documentversionid=42698, 2010).

6 Muller, E. B. & Nisbet, R. M. Survival and production in variable resource environments. *Bulletin of Mathematical Biology* **62**, 1163-1189 (2000).

7 Bourlès, Y. *et al.* Modelling growth and reproduction of the Pacific oyster *Crassostrea gigas*: advances in the oyster-DEB model through application to a coastal pond. *Journal of Sea Research* **62**, 62-71 (2009).

8 Ren, J. S. & Ross, A. H. Environmental influence on mussel growth: a dynamic energy budget model and its application to the greenshell mussel *Perna canaliculus*. *Ecological Modelling* **189**, 347-362 (2005).

9 Rosland, R., Strand, Ø., Alunno-Bruscia, M., Bacher, C. & Strohmeier, T. Applying Dynamic Energy Budget (DEB) theory to simulate growth and bio-energetics of blue mussels under low seston conditions. *Journal of Sea Research* **62**, 49-61 (2009).

10 Teufel, C. Addendum to Staff Report for Consistency Certification CC-035-12, KZO Sea Farms. (State of California - Natural Resources Agency, 2014).

11 Montalto, V., Sarà, G., Ruti, P. M., Dell'Aquila, A. & Helmuth, B. Testing the effects of temporal data resolution on predictions of the effects of climate change on bivalves. *Ecological Modelling* **278**, 1-8 (2014).

12 Kooijman, B., Lika, D., Marques, G., Augustine, S. & Pecquerie, L. *Add-my-pet Dynamic Energy Budget Database. Entry for Mytilus galloprovincialis: http://www.bio.vu.nl/thb/deb/deblab/add_my_pet/entries_web/Mytilus_galloprovincialis_res.html*, <http://www.debtheory.org/wiki/index.php?title=Main_Page> (2014).

13 Stramski, D. *et al.* Relationships between the surface concentration of particulate organic carbon and optical properties in the eastern South Pacific and eastern Atlantic Oceans. *Biogeosciences* **5**, 171-201 (2008).

14 Feldman, G. C., C. R. McClain. (ed N. Kuring, Bailey, S. W.) (NASA Goddard Space Flight Center, 2013).

15 NASA Ocean Biology Processing Group. MODIS-Aqua Level 3 Binned Particulate Organic Carbon Data Version 2014 (2015).

16 Rassweiler, A., Costello, C. & Siegel, D. A. Marine protected areas and the value of spatially optimized fishery management. *Proc. Natl. Acad. Sci. U.S.A.* **109**, 11884-11889 (2012).

17 Pérez, O., Telfer, T. & Ross, L. On the calculation of wave climate for offshore cage culture site selection: a case study in Tenerife (Canary Islands). *Aquacultural Engineering* **29**, 1-21 (2003).

18 Buck, B. H., Ebeling, M. W. & Michler-Cieluch, T. Mussel cultivation as a co-use in offshore wind farms: potential and economic feasibility. *Aquaculture Economics & Management* **14**, 255-281 (2010).

19 ICES. Report of the Working Group on Marine Shellfish Culture (WGMASC). (Sopot, Poland. ICES CM 2012/SSGHIE:15, 2012).

20 Flavin, K., Flavin, N. & Flahive, B. Kelp Farming Manual: A Guide to the Processes, Techniques, and Equipment for Farming Kelp in New England Waters. (Ocean Approved, 2013).

21 Edwards, M. & Watson, L. Aquaculture Explained: Cultivating *Laminaria digitata*. (Irish Sea Fisheries Board, 2011).

22 Dring, M., Edwards, M. & Watson, L. Development and Demonstration of Viable Hatchery and Ongoing Methodologies for Seaweed Species with Identified Commercial Potential. Report No. PBA/SW/07/001, (Marine Institute. http://hdl.handle.net/10793/865, 2013).

23 Broch, O. J. & Slagstad, D. Modelling seasonal growth and composition of the kelp Saccharina latissima. *Journal of Applied Phycology* **24**, 759-776 (2012).

24 Jackson, G. A. Nutrients and production of giant kelp, *Macrocystis pyrifera*, off southern California. *Limnology and Oceanography* **22**, 979-995 (1977).

25 Arkema, K. K. *Consequences of kelp forest structure and dynamics for epiphytes and understory communities* PhD thesis, University of California, Santa Barbara, (2008).

26 Abreu, M. H., Pereira, R. & Sassi, J.-F. in *Marine Algae: Biodiversity, Taxonomy, Environmental Assessment, and Biotechnology* (eds Leonel Pereira & Joao M. Neto) 300-319 (CRC Press, 2014).

27 Chopin, T. Seaweed aquaculture provides diversified products, key ecosystem functions. *Part II. Recent evolution of seaweed industry. Global Aquaculture Advocate* **14**, 24-27 (2012).

28 Song, M., Pham, H. D., Seon, J. & Woo, H. C. Marine brown algae: A conundrum answer for sustainable biofuels production. *Renewable and Sustainable Energy Reviews* **50**, 782-792 (2015).

29 Kiefer, D. & Rensel, J. AquaModel. *System Science Applications, Inc.* <**http://www.aquamodel.net/**> (2016).

30 Rensel, J. J., Kiefer, D. A., Forster, J. R., Woodruff, D. L. & Evans, N. R. Offshore finfish mariculture in the Strait of Juan de Fuca. *Bulletin of Fisheries Research Agency Japan* **19**, 113-129 (2007).

31 Kiefer, D. A., Rensel, J. E., O'Brien, D. W., Fredriksson, D. W. & Irish, J. An ecosystem design for marine aquaculture site selection and operation. NOAA Marine Aquaculture Initiative Program. Final Report. NA08OAR4170859. (System Science Applications, in association with United States Naval Academy and Woods Hole Oceanographic Institution, Available at: http://noaa.aquamodel.net/Documents/AquaModel_NOAA%20NMAI%202011.pdf, 2011).

32 Rensel, J. E., O'Brien, F., Siegrist, Z. & Kiefer, D. A. Tropical open-ocean aquaculture modeling: AquaModel tuning and validation. Prepared for National Marine Fisheries Service, Pacific Islands Regional Office. (System Science Applications, Inc. and Blue Ocean Mariculture LLC, Available at: http://www.aquamodel.net/Downloads/Open%20Ocean%20Validation%20of%20AquaModel%205May2015%20Final%20Report.pdf, 2015).

33 O’Brien, F., Kiefer, D. A. & Rensel, J. E. AquaModel: software for sustainable development of open ocean fish farms. Report prepared for the United States Department of Agriculture. Award No: 2007-33610-18532. (System Science Applications, Inc., Available at: http://usda.aquamodel.net/Downloads/AquaModel%20Final%20Report%20USDA_SBIR_B.pdf, 2011).

34 Kiefer, D. A., Rensel, J. E. J. & O'Brien, F. AquaModel Simulation of Water and Sediment Effects of Fish Mariculture at the Proposed Hubbs-Seaworld Research Institute Offshore Aquaculture Demonstration Project. (Systems Science Applications Rensel Associates Aquatic Sciences, 2008).

35 Schubel, J. R., Monroe, C. Is Offshore Finfish Aquaculture In the Southern California Bight An Idea Whose Time Has Come? , (Aquarium of the Pacific’s Marine Conservation Research Institute, 2008).

36 Zimmerman, R. C. & Kremer, J. N. Episodic nutrient supply to a kelp forest ecosystem in Southern California. *Journal of Marine Research* **42**, 591-604 (1984).

37 Neuheimer, A., Thresher, R., Lyle, J. & Semmens, J. Tolerance limit for fish growth exceeded by warming waters. *Nat Clim Change* **1**, 110-113 (2011).
38 Dasgupta, S. & Thompson, K. R. Comparison of costs of different hybrid striped bass production systems in ponds **SRAC Publication No. 3000.** **http://fisheries.tamu.edu/files/2013/09/SRAC-Publication-No.-3000-Comparison-of-Costs-of-Different-Hybrid-Striped-Bass-Production-Systems-in-Ponds.pdf** (2013).
39 Allen, L. G. & Horn, M. H. *The ecology of marine fishes: California and adjacent waters*. (Univ of California Press, 2006).
40 Moser, H. G. & Watson, W. Distribution and abundance of early life history stages of the California halibut, Paralichthys californicus, and comparison with the fantail sole, Xystreurys liolepis. *California Department of Fish and Game Fish Bulletin* **174**, 31-84 (1990).
41 Maunder, M., Reilly, P., Tanaka, T., Schmidt, G. & Penttila, K. (California Department of Fish and Game, 2011).
42 Pauly, D. & Froese, R. *FishBase. www.fishbase.org*, <www.fishbase.org> (2016).
43 Haugen, C. W. The California Halibut, *Paralichthys californicus*, Resource and Fisheries. *California Department of Fish and Game Fish Bulletin* **174**, 1-476 (1990).
44 von Bertalanffy, L. in *Fundamental aspects of normal and malignant growth* (ed W. W. Nowinski) 137-259 (Elsevier, 1960).
45 Carr, S. D., Capet, X. J., McWilliams, J. C., Pennington, J. T. & Chavez, F. P. The influence of diel vertical migration on zooplankton transport and recruitment in an upwelling region: Estimates from a coupled behavioral□physical model. *Fisheries Oceanography* **17**, 1-15 (2008).
46 Mitarai, S., Siegel, D., Watson, J., Dong, C. & McWilliams, J. Quantifying connectivity in the coastal ocean with application to the Southern California Bight. *Journal of Geophysical Research: Oceans* **114** (2009).
47 Ohlmann, J. C. & Mitarai, S. Lagrangian assessment of simulated surface current dispersion in the coastal ocean. *Geophysical Research Letters* **37** (2010).
48 Watson, J. *et al.* Realized and potential larval connectivity in the Southern California Bight. *Marine Ecology Progress Series* **401**, 31-48 (2010).
49 Simons, R. D., Siegel, D. A. & Brown, K. S. Model sensitivity and robustness in the estimation of larval transport: A study of particle tracking parameters. *Journal of Marine Systems* **119**, 19-29 (2013).
50 Caley, M. *et al.* Recruitment and the local dynamics of open marine populations. *Annual Review of Ecology and Systematics*, 477-500 (1996).
51 Beverton, R. J. & Holt, S. J. *On the dynamics of exploited fish populations*. (Her Majesty's Stationery Office, 1957).
52 Hilborn, R. & Walters, C. J. Quantitative fisheries stock assessment: choice, dynamics and uncertainty. *Reviews in Fish Biology and Fisheries* **2**, 177-178 (1992).
53 White, J. W. Adapting the steepness parameter from stock–recruit curves for use in spatially explicit models. *Fisheries Research* **102**, 330-334 (2010).
54 Myers, R. A., Bowen, K. G. & Barrowman, N. J. Maximum reproductive rate of fish at low population sizes. *Canadian Journal of Fisheries and Aquatic Sciences* **56**, 2404-2419 (1999).
55 Arfken, G. Nonhomogeneous equation Green’s functions. *Mathematical Methods for Physicists, 3rd ed. Academic, Orlando* (1985).
56 Garabedian, P. (Wiley, New York).
57 Cheung, W. W., Pauly, D. & Lam, V. W. Modelling present and climate-shifted distribution of marine fishes and invertebrates. *Fisheries Centre research reports* (2008).
58 Walters, C., Pauly, D. & Christensen, V. Ecospace: Prediction of mesoscale spatial patterns in trophic relationships of exploited ecosystems, with emphasis on the impacts of marine protected areas. *Ecosystems* **2**, 539-554 (1999).
59 Clark, C. *Mathematical bioeconomics: the optimal management of renewable resources*. (John Wiley & Sons, Inc., 1990).

60 White, C., Kendall, B. E., Gaines, S., Siegel, D. A. & Costello, C. Marine reserve effects on fishery profit. *Ecology Letters* **11**, 370-379 (2008).

61 Hannesson, R. A note on the" stock effect". *Marine Resource Economics* **22**, 69-75 (2007).

62 Worm, B. *et al.* Rebuilding global fisheries. *Science* **325**, 578-585 (2009).

63 Smith, M. D. & Wilen, J. E. Economic impacts of marine reserves: the importance of spatial behavior. *Journal of Environmental Economics and Management* **46**, 183-206 (2003).

64 Kaplan, D. M., Botsford, L. W., O'Farrell, M. R., Gaines, S. D. & Jorgensen, S. Model-based assessment of persistence in proposed marine protected area designs. *Ecological Applications* **19**, 433-448 (2009).

65 Little, L. R. *et al.* Different responses to area closures and effort controls for sedentary and migratory harvested species in a multispecies coral reef linefishery. *ICES Journal of Marine Science* **66**, 1931-1941 (2009).

66 Fretwell, S. D. & Calver, J. S. On territorial behavior and other factors influencing habitat distribution in birds. *Acta Biotheoretica* **19**, 37-44 (1969).

67 Branch, T. A. *et al.* Fleet dynamics and fishermen behavior: lessons for fisheries managers. *Canadian Journal of Fisheries and Aquatic Sciences* **63**, 1647-1668 (2006).

68 Gillis, D. M. Ideal free distributions in fleet dynamics: a behavioral perspective on vessel movement in fisheries analysis. *Canadian Journal of Zoology* **81**, 177-187 (2003).

69 Costello, C. & Polasky, S. Optimal harvesting of stochastic spatial resources. *Journal of Environmental Economics and Management* **56**, 1-18 (2008).

70 Brown, C. J. *et al.* Fisheries and biodiversity benefits of using static versus dynamic models for designing marine reserve networks. *Ecosphere* **6**, 1-14 (2015).

71 Gee, K. Offshore wind power development as affected by seascape values on the German North Sea coast. *Land Use Policy* **27**, 185-194 (2010).

72 Haggett, C. Understanding public responses to offshore wind power. *Energy Policy* **39**, 503-510 (2011).

73 Falconer, L., Hunter, D.-C., Telfer, T. C. & Ross, L. G. Visual, seascape and landscape analysis to support coastal aquaculture site selection. *Land Use Policy* **34**, 1-10 (2013).

74 Perez, O., Telfer, T. & Ross, L. Use of GIS-based models for integrating and developing marine fish cages within the tourism industry in Tenerife (Canary Islands). *Coastal Management* **31**, 355-366 (2003).

75 Bernard Brown Associates Ltd. Natural Character and Visual Impact Assessment of Potential Finfish Farming Development. 29 (Environment Waikato Regional Council, Technical Report 2008/24, 2008).

76 Arkema, K. K. *et al.* Coastal habitats shield people and property from sea-level rise and storms. *Nature Climate Change* **3**, 913-918 (2013).

77 California State Park System Statistical Report 2011/12 Fiscal Year. (Statewide Planning Unit Planning Division California State Parks, 2013).

78 NOAA. Fisheries of the Caribbean, Gulf, and South Atlantic; Aquaculture. Rule 81 FR 1761. 1761 -1800 (U.S. Department of Commerce, National Oceanic and Atmospheric Administration. Available at: https://www.gpo.gov/fdsys/pkg/FR-2016-01-13/pdf/2016-00147.pdf, 2016).

79 Klinger, D. & Naylor, R. Searching for solutions in aquaculture: charting a sustainable course. *Annual Review of Environment and Resources* **37**, 247-276 (2012).

80 Lovatelli, A., Aguilar-Manjarrez, J. & Soto, D. *Expanding mariculture farther offshore: technical, environmental, spatial and governance challenges*. (FAO, 2013).

81 Sarà, G., Scilipoti, D., Milazzo, M. & Modica, A. Use of stable isotopes to investigate dispersal of waste from fish farms as a function of hydrodynamics. *Marine Ecology Progress Series* **313**, 261-270 (2006).

82 Ferreira, J., Hawkins, A. & Bricker, S. Management of productivity, environmental effects and profitability of shellfish aquaculture—the Farm Aquaculture Resource Management (FARM) model. *Aquaculture* **264**, 160-174 (2007).

83 Niklitschek, E. J., Soto, D., Lafon, A., Molinet, C. & Toledo, P. Southward expansion of the Chilean salmon industry in the Patagonian Fjords: main environmental challenges. *Reviews in Aquaculture* **5**, 172-195 (2013).
84 Pearson, T. & Rosenberg, R. Macrobenthic succession in relation to organic enrichment and pollution of the marine environment. *Oceanography and Marine Biology - An Annual Review* **16**, 229-311 (1978).
85 Price, C. S. & Morris Jr, J. A. Marine cage culture and the environment: twenty-first century science informing a sustainable industry. *NOAA Technical Memorandum* **NOS NCCOS 164**, doi:10.13140/RG.2.1.2382.9841 (2013).
86 Crawford, C. M., Macleod, C. K. & Mitchell, I. M. Effects of shellfish farming on the benthic environment. *Aquaculture* **224**, 117-140 (2003).
87 Hargrave, B., Holmer, M. & Newcombe, C. Towards a classification of organic enrichment in marine sediments based on biogeochemical indicators. *Marine Pollution Bulletin* **56**, 810-824 (2008).
88 Bondad-Reantaso, M. G. *et al.* Disease and health management in Asian aquaculture. *Veterinary Parasitology* **132**, 249-272 (2005).
89 Murray, A. G. & Peeler, E. J. A framework for understanding the potential for emerging diseases in aquaculture. *Preventive Veterinary Medicine* **67**, 223-235 (2005).
90 Bostock, J. *et al.* Aquaculture: global status and trends. *Philosophical Transactions of the Royal Society B: Biological Sciences* **365**, 2897-2912 (2010).
91 Suttle, C. A. Marine viruses—major players in the global ecosystem. *Nature Reviews Microbiology* **5**, 801-812 (2007).
92 Suttle, C. A. & Chen, F. Mechanisms and rates of decay of marine viruses in seawater. *Applied and Environmental Microbiology* **58**, 3721-3729 (1992).
93 Griffin, R. & Nunn, C. Community structure and the spread of infectious disease in primate social networks. *Evol Ecol* **26**, 779-800, doi:10.1007/s10682-011-9526-2 (2012).
94 Lee, T., Lee, H.-R. & Hwang, K. in *Proceedings of the 2013 Winter Simulation Conference: Simulation: Making Decisions in a Complex World.* 2239-2249 (IEEE Press).
95 Bonacich, P. Factoring and weighting approaches to status scores and clique identification. *Journal of Mathematical Sociology* **2**, 113-120 (1972).
96 Newman, M. E. in *The new Palgrave encyclopedia of economics* (eds L.E. Blume & S.N Durlauf) (Palgrave Macmillan, 2008).
97 Haamer, J. Improving water quality in a eutrophied fjord system with mussel farming. *Ambio* **25**, 356-362 (1996).
98 NASA Ocean Biology Processing Group. MODIS-Aqua Level 3 Mapped Photosynthetically Available Radiation Data Version 2014 (2015).
99 Watson, L., O' Mahony, F., Edwards, M., Dring, M.L., & Werner, A. The Economics of Seaweed Aquaculture in Ireland *Laminaria digitata* and *Palmaria palmata*. Irish Sea Fisheries Board (2012).
