# Supplementary material for: Marine spatial planning makes room for offshore aquaculture in crowded coastal waters
Source: Nat Commun. 2018 Mar 5;9:945. doi: 10.1038/s41467-018-03249-1 (PMC5838171; doi:10.1038/s41467-018-03249-1)
Supplement: Supplementary file 3 — Description of Additional Supplementary Files [file 41467_2018_3249_MOESM3_ESM.pdf]

Description of Additional Supplementary Files

File Name: Supplementary Data 1
Description: **Spatial data layers.** Source, year and weblink for spatial data layers used in analyses and by component models.

File Name: Supplementary Data 2
Description: **Model parameters and variables**. Model parameters and other variables used in component models, including values and supporting references.
